# Setting up a pragmatic clinical trial in a low-resource setting: A qualitative assessment of GoLBeT, a trial of podoconiosis management in Northern Ethiopia

**DOI:** 10.1371/journal.pntd.0009582

**Published:** 2021-07-28

**Authors:** Astrid C. Erber, Victoria Ewing, Mark Turner, Meseret Molla, Gharib Murbe, Fikre Enquoselassie, Gail Davey, Trudie Lang

**Affiliations:** 1 Centre for Tropical Medicine and Global Health, Nuffield Department of Medicine, University of Oxford, Oxford, United Kingdom; 2 Department of Epidemiology, Center for Public Health, Medical University of Vienna, Vienna, Austria; 3 Office for National Statistics, Government Buildings, Newport, United Kingdom; 4 School of Public Health, Addis Ababa University, Addis Ababa, Ethiopia; 5 Centre for Global Health Research, Brighton & Sussex Medical School, University of Sussex, Brighton, United Kingdom; Federal University of Ceará, Fortaleza, Brazil, BRAZIL

## Abstract

**Background:**

Clinical trials are often perceived as being expensive, difficult and beyond the capacity of healthcare workers in low-resource settings. However, in order to improve healthcare coverage, the World Health Organization (WHO) World Health Report 2013 stated that all countries need to become generators as well as recipients of data. This study is a methodological examination of the steps and processes involved in setting up the Gojjam Lymphoedema Best Practice Trial (GoLBeT; ISRCTN67805210), a highly pragmatic clinical trial conducted in northern Ethiopia. Challenges to the trial and strategies used to deal with them were explored, together with the reasons for delays.

**Methodology and principal findings:**

Qualitative research methods were used to analyse emails and reports from the period between trial inception and recruitment. This analysis was complemented by interviews with key informants from the trial operational team. The Global Health Research Process Map was used as a framework against which to compare the steps involved in setting up the trial. A mini-group discussion was conducted with the trial operational team after study completion for reflection and further recommendations.

This study showed that the key areas of difficulty in setting up and planning this trial were: the study design, that is, deciding on the study endpoint, where and how best to measure it, and assuring statistical power; recruitment and appropriate training of staff; planning for data quality; and gaining regulatory approvals. Collaboration, for example with statisticians, the trial steering committee, the study monitors, and members of the local community was essential to successfully setting up the trial.

**Conclusions and significance:**

Lessons learnt from this trial might guide others planning pragmatic trials in settings where research is not common, allowing them to anticipate possible challenges and address them through trial design, planning and operational delivery. We also hope that this example might encourage similar pragmatic studies to be undertaken. Such studies are rarely undertaken or locally led, but are an accessible and efficient way to drive improved outcomes in public health.

## Author summary

Podoconiosis is a disease of the lymphatic vessels of the lower extremities, caused by chronic exposure to irritant soils. It results in leg swelling, commonly complicated by acute dermatolymphangioadenitis (ADLA, or ‘acute attacks’), characterised by severe pain, fever and disability. The Gojjam Lymphoedema Best Practice Trial (GoLBeT) trial is a successfully completed randomised controlled trial of podoconiosis treatment that was conducted in the highlands of northern Ethiopia. In this study we report the steps involved in the setup of this trial, from inception to recruitment. The challenges faced are discussed, along with recommendations on how these could be overcome.

## Introduction

Clinical trials are often perceived as being expensive, difficult and beyond the capacity of healthcare workers in low-resource settings. Moreover, in such settings, it is a common view that clinical trials are conducted by ‘foreigners’, and there is limited awareness that trials can assess more than new drugs and vaccines. The Gojjam Lymphoedema Best Practice Trial (GoLBeT) is a successfully completed, pragmatic, randomised controlled trial of podoconiosis treatment that was conducted in the highlands of northern Ethiopia [1–3]. Podoconiosis is a non-parasitic form of leg swelling (lymphoedema), which, within Ethiopia, is currently being treated on a relatively small scale by NGOs using simple methods and locally available materials [4]. GoLBeT is an important and unusual clinical trial because it assessed a highly pragmatic intervention (a simple foot hygiene package) that is not a drug or vaccine. The primary outcome was incidence of ‘acute attacks’ (painful, disabling episodes of inflammation in the swollen leg). In general, pragmatic trials aim to assess real-world effectiveness in broad patient groups by including a population relevant for the intervention, thereby maximising external validity and informing practice [5]. There are many calls for more disease management trials, like GoLBeT, that measure whether simple and locally available interventions can improve health outcomes [6,7]. Such trials might compare new ways of using existing therapies, train healthcare workers in new approaches, or assess whether patients are managed better at home or in hospital (e.g., [8]).

There are not enough locally led clinical trials in resource-limited settings such as Ethiopia, and there are many barriers to health workers becoming researchers in these settings [9,10]. The World Health Organization (WHO) World Health Report 2013 [11] made a clear call for such countries to become data generators, and not only the recipients of data, to facilitate real improvements in health outcomes in these regions. Although there are comprehensive programmes to encourage clinical trial methodology research (e.g. the Medical Research Council’s Hubs for Trials Methodology Research [12] in Europe, and the Clinical Trials Transformation Initiative [13]), insufficient research has focussed on operations and processes, and very few studies have examined the specific issues facing clinical trials in resource-limited settings. Little is therefore known about the true barriers that researchers face when setting up a trial in a place where research is uncommon.

Using an action research approach [14,15], we examined the process of setting up GoLBeT, a highly pragmatic randomised trial, to identify the challenges faced by the team and report how these challenges were overcome. Action research ‘sets out to explicitly study something in order to change and improve it. …*It most often arises from an unsatisfactory situation that those most affected wish to alter for the better’* ([14], p.1). We aimed to learn from the process of setting up this study, and to encourage and benefit future research teams. We hope that this example will act as a guide for others to follow, and demonstrate that important pragmatic trials can be achieved and successfully completed in low-resource settings. The processes outlined are adaptable to any health research study conducted in a similar setting.

## Methods

### Ethics statement

This work is a minimal risk methodology study process evaluation where the only participants are the study team. Therefore this does not come under the definitions of health research into human subjects. The protocol for this study was approved by ethics committees in Oxford, Brighton, and Ethiopia.

### Study design

This research methodology study documented the steps undertaken in the process of trial set-up for the GoLBeT study (ISRCTN67805210) from inception to recruitment, drawing comparisons with The Global Health Research Process Map (the most current, interactive version is available at: https://processmap.tghn.org) [16]. The Global Health Research Process Map outlines the steps required to set up a clinical trial, and was designed as a resource for The Global Health Network website (https://tghn.org) [16–18]. It was selected as a valid framework against which the real issues that occurred in GoLBeT could be compared and considered.

Discussions about GoLBeT first took place in September 2011, and this study documents the steps of set-up from then until recruitment in January 2015.

Qualitative research methods using a framework analysis approach [19,20] were used over three stages. As main informants, three key members of the trial team were purposively chosen: The Principal Investigator, the Trial Coordinator and the Trial Data Manager. First, trial-related emails and reports from the period between the inception of GoLBeT and recruitment were analysed. Second, telephone interviews were conducted with two members of the trial team and one structured, open-ended interview questionnaire was completed by a member of the team with whom a clear telephone line could not be established. This step was necessary to complement and clarify findings from the document analysis conducted in the previous step. Third, a mini-group discussion was conducted immediately after the end-of-study meeting with all three key members of the trial team, to provide an opportunity to retrospectively reflect upon the trial set-up and initiation phase, and to provide recommendations from that perspective.

In summary, trial activities conducted were linked to each step on the Global Health Research Process Map.

The individuals involved in these activities, challenges faced, and methods used to resolve these challenges were identified. The comparison with the Global Health Research Process Map allowed further observations to be made about the order in which steps were undertaken and in which issues arose; how long they took to resolve; and the impact that this had on the start of the study, with a view to providing recommendations for future trials.

The first two stages were conducted by two social scientists experienced in qualitative research, and the third one by a clinical researcher trained in qualitative research methods, all working closely with the trial team over several feedback rounds. This action research approach ensured triangulation of findings, and discussion of discrepancies in analysis and interpretation.

The protocol for this methodology study was presented at the 2^nd^ Clinical Trials Methodology Conference: Methodology Matters (Edinburgh, UK, 18–19 November 2013) [21].

### Analysis of emails and study reports

**Fig 1:**
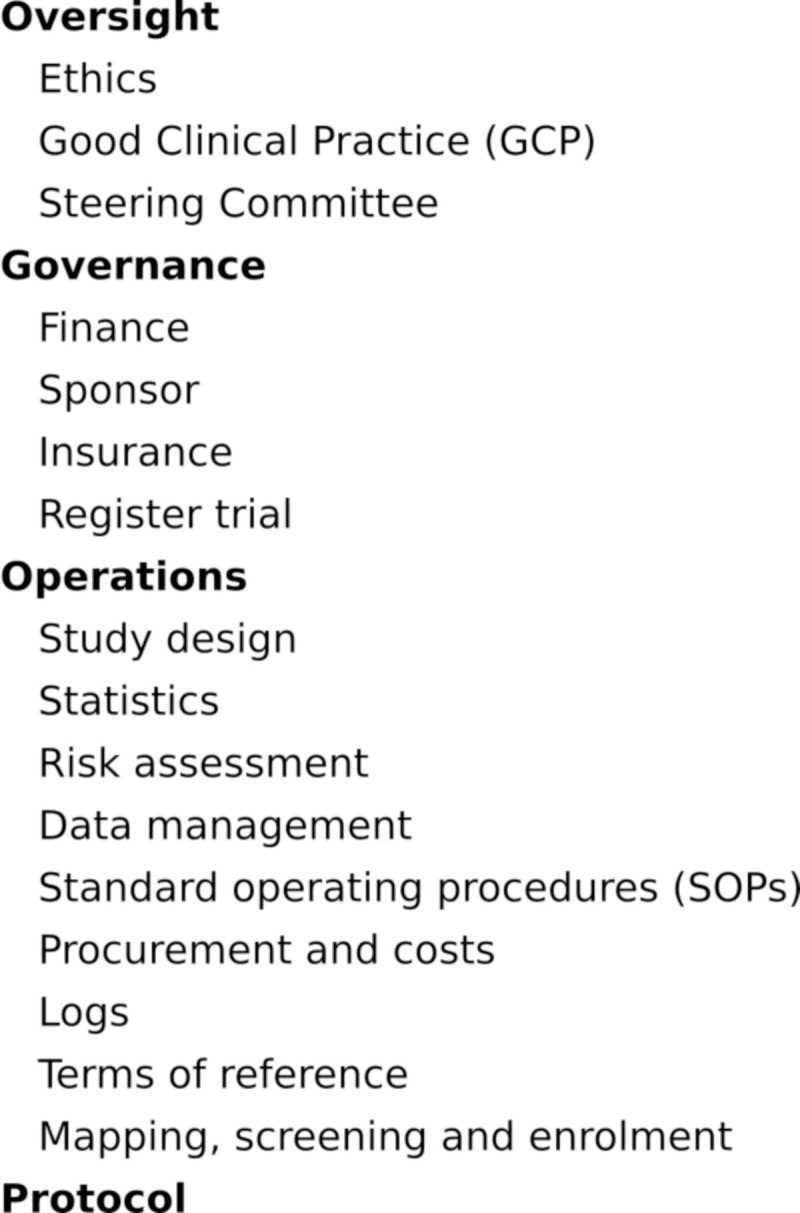
NVivo node tree. The tree consists of a hierarchy of parent nodes and nodes for organizing text passages from data (coding). Parent nodes are in bold. For the parent node *Operations*, key nodes are shown only.

Approximately 750 emails and 25 study coordinator reports relating to GoLBeT were imported into NVivo 10 (QSR International) for coding and analysis. These dated from the trial inception in September 2011 to recruitment in December 2014 and January 2015. The emails had been sent between key trial team members, stakeholders, collaborators and advisers. A framework analysis approach [20] was used to analyse the data, which were coded using a framework based on the five process areas of the Global Health Research Process Map: oversight; governance; operations; protocol; and intervention. Nodes used to code the data under each of these process areas were also broadly based on the Global Health Research Process Map, but adapted as necessary according to the findings from the trial. Three additional categories were developed: key people and organisations (e.g., trial team, sponsors, statisticians, trial monitors, institutional review boards [IRBs], regulators, and community leaders); actions (e.g., submitting documents, meetings conducted, and approvals and agreements; and issues (e.g., lack of experience, schedule and delays, equipment and technical). The NVivo node tree is shown in Fig 1.

Matrix queries were run in NVivo to explore the intersection of different nodes. For example, a query was run for ‘Ethics by all nodes’, to see where this topic node intersected with key people, different actions and issues, and with other topic nodes, such as ‘Protocol’. The matrix query therefore showed the various contexts in which the topic of ethics appeared in GoLBeT.

The matrix query results for all topic nodes were then assigned to the 41 steps of the Global Health Research Process Map, so that content showing the topics, actions, people, and issues associated with each step was visible. The dates of key events were also recorded.

### Interviews and questionnaire

The Principal Investigator, the Trial Coordinator and the Trial Data Manager as key members of the trial team were further consulted using interviews or an open-ended questionnaire in November and December 2014, immediately before the start of recruitment. Interview guides and questions were developed for each, based on the role of the informant and issues that had been identified during earlier data collection. Telephone interviews were conducted separately with two members of the trial team and these were recorded and transcribed verbatim. Due to challenges in obtaining a clear connection to Ethiopia, one trial team member was asked to complete an open-ended questionnaire rather than an interview. The interview transcripts, completed questionnaire, and notes taken after an earlier teleconference with the Trial Coordinator and Data Manager were all coded according to the 41 Global Health Research Process Map steps. This additional coding complemented and clarified data from the emails and the reports, and provided rich information that was often absent from short emails.

Summaries of what occurred at each step of the Global Health Research Process Map were then prepared. These summaries provided an account of setting up this pragmatic treatment trial, and showed what the challenges were and how they were resolved. The key dates in the summaries also provided a timeframe for the different steps, showing when they occurred and how long they took.

### Mini-group discussion

A mini-group discussion [22–24] with the three key members of the trial team who had been consulted in the last step was conducted immediately after the end-of-study meeting in May 2017 in Addis Ababa, Ethiopia. An interview topic guide was developed based on previous analysis results, and participants were also encouraged to discuss new themes that had not emerged earlier. The group discussion was audio recorded, and detailed notes taken by the facilitator. The audio recording was transcribed verbatim and coded in NVivo for thematic analysis, supported by the notes taken. The purpose of this final stage was to reflect upon the setting up of GoLBeT from that later perspective, with a view to providing recommendations for future researchers.

## Results

The results below describe the methodological process of setting up GoLBeT by first, outlining the overall differences to the Global Health Research Process Map and second, detailing the key steps required in setting up GoLBeT, thereby also assigning a time frame to the different elements. Subsequently, this section describes the substantive issues that were faced and strategies used to deal with them, as well as recommendations given by the trial team after trial completion.

### Overall differences between the process of setting up GoLBeT and the Global Health Research Process Map

**Fig 2:**
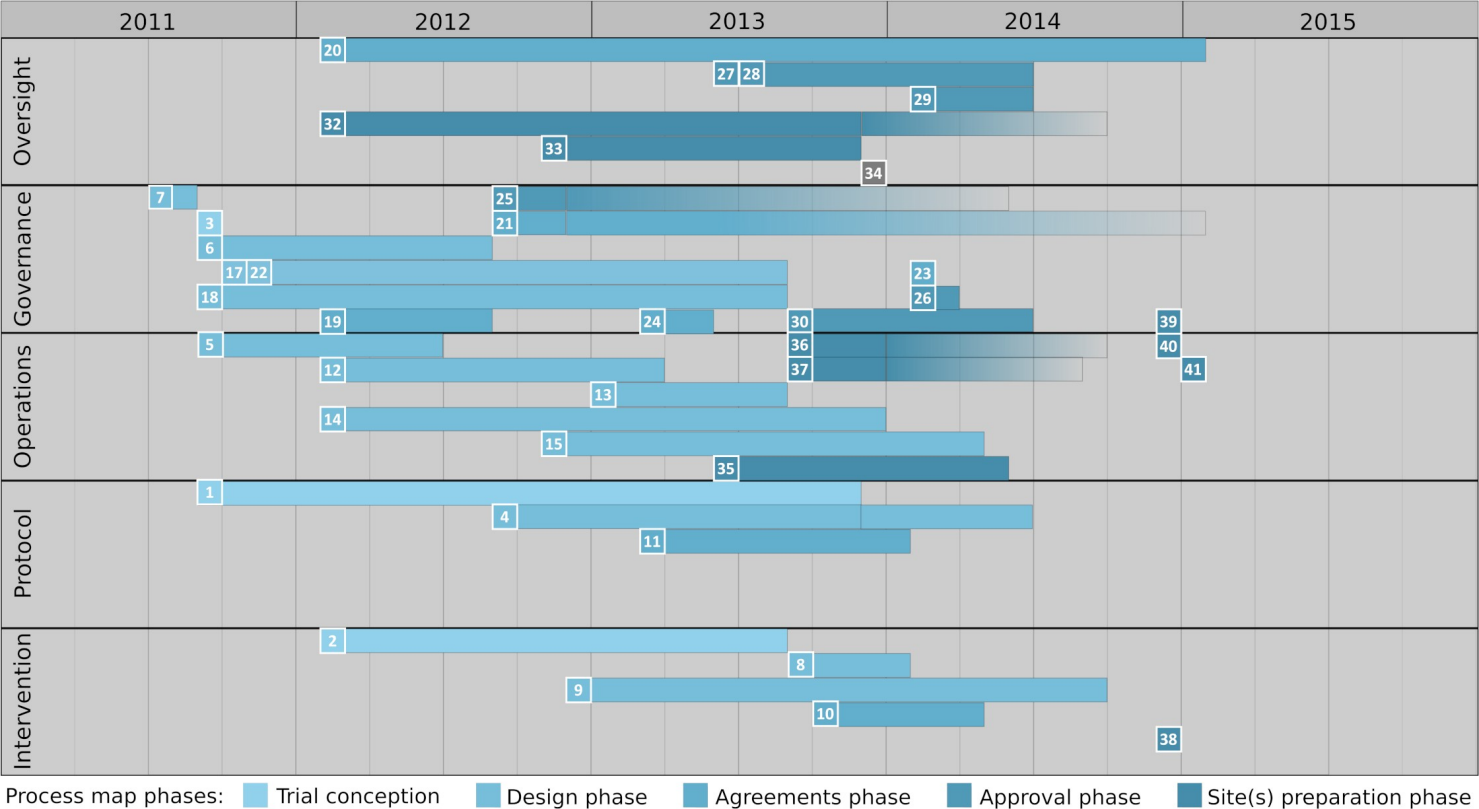
GoLBeT initiation and set-up process. The different steps identified are grouped into the following categories: oversight, governance, operations, protocol and intervention. The steps, grouped by categories, are: Category *Oversight*: 20 –Peer Review, 27/28 –Submit to Institutional Review Board(s)/Submit to Ethics Review, 29 –Submit to Clinical Trial Authority Review, 32 –Data Safety Monitoring Board (DSMB) needed?, 33 –Steering Committee, 34 –Endpoint Review needed?. Category *Governance*: 3 –Characterise Study and Identify Regulations, 6—Identify Sponsor, 7 –Identify Source of Funding, 17 –Identify Insurance Required, 18 –Set Budget, 19 –Funding Proposal, 21 –Secure Funding, 22 –Organise Appropriate Legal Cover, 23 –Sponsor Approval of Protocol, 24 –Contracts Agreed, 25 –Register Clinical Trial, 26 –Regulatory Approvals Submission, 30 –Assemble Essential Documents in Study Master File, 39 –Study Initiation Visit. Category *Operations*: 5 –Study Design, 12 –Consider Study Population, Sample Size and Trial Statistics, 13 –Consider Data Management Requirements, 14 –Risk Assessment, 15 –Develop Quality Assurance Plan, 35 –Set up Clinical Data Management System, 36 –Finalise SOPs, 37 –Complete Training in SOPs & GCP, 40 –Information Sessions for Study Staff, 41 –Begin Recruitment. Category *Protocol*: 1 –Develop Research Question, 4 –Develop Study Protocol, 11 –Finalise Protocol. Send to Sponsor. Category *Intervention*: 2 –What is the Intervention?, 8 –Intervention and Laboratory Organisation, Supply and Logistics, 9 –Develop Pharmacovigilance / Safety Reporting Plan, 10 –Finalise Intervention and Laboratory Requirements, 38 –Are Study Supplies Ready?. Miscellaneous: 16 –Develop Trial Management System, 31 –Setup Oversight Committees Stated in QA Plan. A detailed description of each step can be found in S1 Text: GoLBeT initiation and set-up. Bars indicate the approximate duration of each step and colours correspond to the phases in the Global Health Research Process Map [16] that these activitities were assigned to. Fading bars indicate that processes, rather than having a defined end point, were going on for longer.

The process of setting up GoLBeT (Fig 2) differed from the apparently linear flow of the Global Health Research Process Map (https://processmap.tghn.org).

In GoLBeT, there was a much greater overlap of steps than in the Global Health Research Process Map, in which steps proceed in a sequential, stepwise manner. In GoLBeT, steps often ran alongside each other, and were frequently developed together, along with key documents. For example, the GoLBeT funding proposal was developed alongside the budget. At the same time, the study population, study design, details of the intervention, and the research question were all considered together, as they were interrelated, and changes in these elements were reflected in the funding proposal and budget. It was often difficult to distinguish clear, sequential steps, due to steps being thought about concurrently.

Notably, key events sometimes initiated several steps at around the same time. For example, the acceptance of the proposal for funding led to activities needed to secure funding contracts, to register the trial, and to start the development of the protocol based on the funding proposal. Delays in key events may have delayed the entire trial. For example, there were several delays in gaining final approvals for the GoLBeT protocol, as outlined below, which delayed the start of the trial.

Steps often took appreciable time to be completed and had to be revisited later before they could be resolved: this sometimes had a rate-limiting effect on future steps. For example, the Global Health Research Process Map step ‘organise appropriate legal cover’ was revisited when an insurance letter was needed while assembling the documents for the Study Master File.

It was often difficult to ascertain when a particular Global Health Research Process Map step began and ended in GoLBeT. However, steps were often thought about early in the set-up process, and earlier than the Global Health Research Process Map might suggest with regard to other steps. For example, the ‘consider data management requirements’ step began before the ‘consider study population, sample size and trial statistics’ step had been finalised, so that the correct technical equipment could be in place in good time.

Other GoLBeT set-up steps were conducted in a different order than they appear in the Global Health Research Process Map. For example, ‘submit to IRB(s)’ came before ‘regulatory approvals submission’ in Ethiopia. The Trial Steering Committee (TSC) was also created much earlier in the whole process, helping with protocol development and to define the study population. The requirement for a Data Safety Monitoring Board (DSMB) was also considered near to the beginning of the GoLBeT set-up process.

### Key steps required in setting up GoLBeT

This section is a simplified account of key events in setting up GoLBeT, from trial inception to participant recruitment. It explains the steps that were occurring at around the time of these key events, and where possible, start dates for these steps, corresponding to the GoLBeT initiation and set-up process (Fig 2). The terms used in this section relate to the titles of the Global Health Research Process Map steps.

### Funding proposal preparation, submission and approval

In September 2011, the source of funding had been identified and an outline proposal was submitted, explaining the purpose of the trial. In the outline proposal, the study was characterised and early drafts for the budget, research question and study design were presented.

The full funding proposal was submitted in February 2012. This addressed the issues of study design, study population, and the precise intervention, which had been queried by the funders. The funders had also suggested ongoing statistical support, and therefore a Co-Investigator arranged for peer review and trial monitoring by the Kemri-Wellcome Programme in Kenya. The full proposal also discussed risk assessment, explaining for the first time why a DSMB was not thought necessary.

The full proposal was provisionally accepted for funding in September 2012, on the conditions that the trial team provided further information and registered the trial. Funding approval also led to the development of the draft protocol. In the coming months, the trial team started to consider quality assurance, by researching standardised definitions for ‘acute attacks’ in podoconiosis, and how to accurately measure the frequency of these episodes (November 2012). Data management requirements were also dealt with, by setting up technical equipment such as computers, servers, and internet access (January 2013).

### Trial Steering Committee (TSC)

In March 2013, the TSC was approved by the funders, and the TSC held discussions using Skype (Microsoft, United States) for the first time, to start to finalise the protocol. The TSC gave considerable input into many aspects, such as the statistics and sample size to be used, and advised on how to explain changes in the sample size to the funders. The TSC also contributed to the development of the pharmacovigilance plan, with regard to expedited reporting for serious adverse events. At this time, contracts were also being arranged, including terms of reference for the TSC and subcontracts for the International Orthodox Christian Charities (IOCC) (United States; the NGO responsible for project management), and the Kenyan study monitors.

### Site visit and ‘study walk-through’ process

There was a major site visit in April 2013, during which a ‘study walk-through’ process was undertaken by the trial team. Key operational questions were addressed by considering every study step, in the reality of the trial setting and with the staff involved. Through this process, key issues were addressed, such as the definition of the primary endpoint and how it was to be measured. Other issues were also considered, such as whether participants could complete a diary card, and the likelihood of participants in different trial arms sharing their intervention. This visit allowed the funding application to be turned into a final protocol.

### Submission to IRBs and ethics committees

The TSC approved the protocol for submission to IRBs and ethics committees in June 2013, and the team started to put together the submission package. The submission package included the protocol, draft baseline questionnaires, and an explanation that the patient information sheet and consent form would follow after the Rapid Ethical Assessment (REA). REA is a form of rapid assessment, conducted at the beginning of a research project, to understand the communities’ perception of the research and engage them in the process. Aims include general mapping of the ethical terrain of a research setting, that is, reconciling universal ethical guidance with specific research contexts. More specifically, REA could aim at exploring challenges associated with approaching communities and gaining informed consent prior to recruitment of participants [25–27]. The REA took place in August 2013 and was an essential part of understanding the concerns of the local communities; results have been published [28].

### Standard Operating Procedures (SOPs)

In September 2013, members of the trial team visited Kilifi, Kenya, to work with members of the Kilifi Clinical Trials Facility on the standard operating procedures (SOPs) for GoLBeT. The SOPs were central to quality assurance, and the trial team needed to understand procedures well to avoid inconsistencies in the SOPs, which could cause protocol deviations. There were SOPs for all trial procedures, including measurement, treatment (once treatment had been standardised), and serious adverse events (the SOP for serious adverse events described the safety reporting plan). SOPs for fieldworkers were prioritised and were the first to be finalised, so that trial staff could be recruited and trained in October 2013, to ensure adherence to Good Clinical Practice (GCP) principles, such as ethics and data quality. REA was used to inform the design of the SOPs for the consent process. Data management SOPs were developed alongside the design of the Case Report Form and the OpenClinica (OpenClinica LLC, United States) database. The trial team learned about storage of clinical supplies and intervention products. While awaiting ethical approvals, the trial coordinator discussed the supply of the intervention products with the manufacturers.

### Ethics approvals and study start

The time taken to gain approvals was already delaying the start of the trial. However, ethical approval was obtained from the IRB in January 2014. This led to the ethics committee at the sponsoring institution confirming its own approval, and meant that a submission could now be made to the national regulator and also to the national research ethics committee in Ethiopia.

Ethics approval required further minor amendments to the protocol, and to related documents such as the SOPs, patient information sheet and consent form. For example, the minimum age of participation was raised from 16 years to 18 years. The trial registration information also needed to be updated. This process took longer than expected, the trial gained its final national-level approvals in July 2014.

Delays in gaining national-level approvals meant that screening would fall in the rainy season (July–September). It was decided that screening would be delayed until October 2014, to avoid difficulties for patients travelling during the rainy season. By December 2014, screening was coming to an end, and the study initiation visit took place just before enrolment. At this visit, there were information sessions to check that study staff were ready, and a further check was made on study supplies. Enrolment ended in January 2015 and was quickly followed by randomisation and trial initiation, approximately one year later than originally planned.

### Substantive issues

This section considers areas in which challenges were encountered in setting up GoLBeT, including a study design in order to achieve statistical power, ensuring data quality, gaining approvals, and dealing with delays. Trial team feedback and strategies that they used to deal with these challenges are also explored.

### Study design

Ensuring that the trial had sufficient statistical power to answer the research question was crucial to the trial’s success and required advice from groups such as the funders, or research groups at the Kemri-Wellcome Programme in Kenya. Early feedback from the funders on the outline proposal queried the ‘stepped wedge’ trial design and requested that the trial team obtain further advice on study design:

“*That was while putting together the full proposal*, *and the comments were very helpful and I’m sure the trial is better as a result of them”–GoLBeT team member 1*

This feedback ultimately improved statistical power, since advice from independent statisticians led to a simpler trial design, with a single primary outcome. An ‘intensive treatment’ arm was also dropped, because this would have reduced statistical power and been impractical to roll out after the trial had finished.

The TSC also advised that the original sample size calculation had not adequately taken clustered data into account, leading to recalculation and dropping of the ability to analyse outcome by four confounders.

The final trial design is presented in the GoLBeT protocol [2] and the published results [3].

### Staff recruitment

Recruitment of suitable staff with the right experience and education, and matching these to the previously defined criteria for trial staff roles was brought up as an important issue.

“*The criteria for recruiting … staff [were] decided by our research team while we visit[ed] the study site last year*. *… There are many challenges about recruiting staff at the field level while also at the head office level*. *… This is very common in developing countries*. *[It is difficult to find people with the right experience] but there are also other issues which make it a challenge*. *The ones that we recruited*, *[for example] this data collector/data entry clerk …*, *our criteria do not [fit]*. *They are more experienced*. *So*, *for data collection*, *for instance*, *you may not need a degree*.*”–*GoLBeT team member 3

### Patient recruitment and retention

REA suggested that members of the local communities would have an important role to play in participant recruitment and retention and that strategies to ensure retention would be necessary. The detailed results of the REA have been published previously [28].

“*That [REA] was extremely beneficial*, *it always is*, *and it’s time really well spent before a major study or a particularly sensitive study*, *or in a community where not much research has taken place*.*”–*GoLBeT team member 1

“In general, it [the REA] really helped in designing the information sheet and consent forms as well as [to] improve our understanding of the norms and cultures of the communities."–GoLBeT team member 2

The REA showed that there were perhaps fewer concerns about participating in the trial than initially thought, but that information provided about the trial, regarding the differences between research and treatment, needed to be presented more clearly. It established the methods by which information related to the trial should be presented to this community. The REA also showed that the way that the informed consent process, randomisation, and delayed treatment were described was also important, if potential participants were to feel comfortable about being enrolled. Finally, the detailed local knowledge of health workers during the mapping and screening process enabled the trial team to locate, and subsequently to enrol and retain, sufficient numbers of patients with podoconiosis.

“*The most important thing here is our fieldworkers are recruited from the kebele [local administrative area] that we are working in*, *and [that] they know the people very well*.*”–*GoLBeT team member 3

### Data quality

Various groups helped to ensure data quality in GoLBeT. In particular, the involvement of the Kilifi Clinical Trials Facility in Kenya was crucial in monitoring the trial and advising the GoLBeT team. The experience of the Kilifi Clinical Trials Facility in conducting clinical trials helped to build capacity among the Ethiopian trial team, who were experienced health team members, but relatively inexperienced in conducting trials.

“*They had the capacity and were very*, *very interested*, *it being a very pragmatic trial*, *and there was this really nice thing about South to South [between-country] support–I really liked that and I think so did the folks in Ethiopia and Kenya*.*”–*GoLBeT team member 1

The Kilifi Clinical Trials Facility helped the GoLBeT team to develop SOPs, which are central to data quality and consistency.

“*That is useful because they have even commented [the] most important things when we sent these documents [SOPs] [to] them*.*”–*GoLBeT team member 3

The Kilifi Clinical Trials Facility also trained the data manager on how to use OpenClinica (OpenClinica LLC, United States), a widely used open-source software for clinical research, and helped with the design of data management documents and systems, including the Case Report Form, the OpenClinica templates, and the database itself. A backup server at the Kilifi facility allowed the monitors to have easy access to trial data and provided security in case the server in Addis Ababa, Ethiopia, failed. Of note, OpenClinica does not support the Ethiopian 13-months calendar format (GoLBeT team member 3, personal communication), posing a challenge.

The trial team needed to standardise the definition and measurement of acute episodes of podoconiosis. The GoLBeT team consulted local podoconiosis treatment groups, as well as the international literature, before agreeing on definitions and procedures to use for the trial, since standard practices are essential for data quality. A well-designed patient diary was needed to record ‘acute attacks’ for the primary outcome, and this required piloting and a feasibility study.

*“Yes*, *that was also something that was brought up by the Trial Steering Committee*. *This is your main outcome*. *You’ve got to be sure that*, *A*, *people can record these events accurately and*, *B*, *they represent what you think they represent*.*”–*GoLBeT team member 1

A local artist drew images of podoconiosis for the diary, helping to ensure that the diary was relevant to the community and able to be used easily and accurately. Well-trained fieldworkers were important for data quality, and translated SOPs were used for training purposes. These SOPs needed to adhere to GCP without being overly complicated, which would risk inaccuracies and protocol deviations, and this required substantial development work. The Kilifi Clinical Trials Facility supported the trial team in conducting fieldworker training, before monitoring the start of the trial and the trial data itself.

### Approvals and delays

GoLBeT started approximately one year later than initially planned, and this was largely due to delays in gaining ethical and regulatory approval from the necessary committees. The sequence required for submission was important because approvals had to be gained consecutively rather than in parallel. For example, departmental approval was needed first from the School of Public Health (Addis Ababa University, Addis Ababa, Ethiopia), before applying to the Addis Ababa University Institutional Review Board (AAU-IRB). After gaining approval from the IRB and the Research Governance and Ethics Committee at the UK sponsoring institution, the process could continue with the national regulator and the national ethics review committee in Ethiopia. This sequence is likely to vary from one country to another.

GoLBeT involved an intervention product, Whitfield’s ointment, that is publicly available, widely used, and is generally well tolerated [29]. Nonetheless, several debates took place about whether a DSMB was needed. The AAU-IRB appeared to demand this, but the experience of Co-Investigators, the TSC, and the Kilifi Clinical Trials Facility monitors eventually persuaded AAU-IRB that a Local Safety Monitor and the safety reporting plan would fulfil GCP requirements. This suggested that official bodies may, at times, have an overly cautious approach to application of GCP, relative to the risk and complexity of the trial. GCP is supposed to be applied according to the requirements of the study. However, in practice, many trials are required to apply GCP in full, which can be inappropriate and make trials unfeasible [30]. There were concerns that *GoLBeT* would require the same level of monitoring that would be applied to a high-risk trial of a new, investigational product.

“It serves no one a good purpose to just directly transplant from a clinical trial of a much more high-risk intervention. To just take exactly the same procedures is really not helpful all round, so it makes sense to look very carefully at the intervention proposed and to adapt the monitoring and then the safety procedures to the level of risk.”–GoLBeT team member 1

There was also a debate about the need for the trial itself, when the AAU-IRB argued that the treatment was already known to be effective. The trial investigators explained that there was no trial-based evidence for the efficacy of the intervention in podoconiosis. However, this debate suggested a possible lack of awareness of the value of clinical trials among the authorities. This was also suggested by a comment from a team member:

“*Within Ethiopia*, *there are people that know that it looks like it works and so there’s people that think it [the intervention] should just go ahead and we don’t need evidence*.*”–*GoLBeT team member 1

Despite the nature of the intervention product, regulatory approval was still required, together with permission to purchase the product from manufacturers. The trial team were aware of this, and so this did not cause delays, but the regulator was initially uncertain about whether purchase approval would be needed. More challenging was the situation around the National Research Ethics Review Committee, which was disbanded in the weeks following submission of the GoLBeT proposal and not re-formed for several months. A compromise was negotiated, in which a reviewer and the new committee chair would approve the trial, but not before the trial had been delayed further. This illustrated the possibility that official national bodies, required for approval, may not always be fully functioning. The delays had a knock-on effect, with the trial team needing to delay screening and enrolment until after the rainy season. Ultimately, the trial team needed to be very flexible in dealing with the delays.

“*The delay with the ethical approval*, *well*, *this happens and the main thing is that we productively used the time and nobody loses heart about it really*, *… this is just part of running studies in such settings*.*”–*GoLBeT team member 1

“*We deployed data collectors exactly one year after training them*. *Of course*, *we had to retrain them in September 2014*. *There’s phone calls every day to check if we’re starting*. *And*, *we also lost some of the previously trained fieldworkers and had to replace them*. *This will also have effect on follow-up data collection during the next rainy season*. *…If we’d started according to plan*, *we’d have been able to avoid it*.*”–*GoLBeT team member 2

The trial team explained to fieldworkers why there had been delays, and these fieldworkers needed to be retrained. Furthermore, trial funding arrangements needed to be flexible enough to allow for a no-cost extension, which was granted in response to a request from the Principal Investigator.

### Reflections from the trial team

Lessons learned and recommendations drawn from this work to guide the planning and set-up of future studies were discussed during a mini-group discussion immediately after the end-of-study meeting. The discussion concluded that initiating and maintaining collaborations would be the most important factor in the success of a pragmatic trial such as GoLBeT. The second key factor would be to ensure comprehensive training for staff, in order to facilitate optimum delivery of the trial and assure data quality. The third key element, community engagement, was considered to be critically important for pragmatic trials in settings where research is novel. In GoLBeT, the approach of REA was used, which the team felt was highly relevant, appropriate and feasible in the Ethiopian context [25,31]. It was clear that the team felt that this approach improved patient recruitment and retention in GoLBeT.

Further recommendations from the team included: being aware of cultural factors and language used; conducting a process evaluation, including qualitative components; maintaining active contact with the ethics committees; and using electronic data collection devices in combination with OpenClinica for data management.

Cultural awareness among staff, as noted previously, was seen as essential, particularly given the remote setting in which the trial was conducted, and the differences between this area and where trial staff were from. One recommendation given was that the team leader should accompany the team when first approaching a remote area:

“*The other key solution to go remotely*, *to get data*, *in particular in screening and enrolment*, *[is] that you should also be part of the system*. *For the first time*, *for instance*, *if there is a very far and remote area*, *for the first time you should go with the team*. *After that*, *if you send them [without travelling yourself]*, *that is ok*, *you will be in that system*. *Do you get my point*? *This is a very critical thing*.*”–*GoLBeT Team member 3

Taking into account the use, and understanding, of specific local terms were also seen as crucial. Including a team member who spoke the local dialect was essential when providing information as part of the informed consent process.

On several occasions, gender-related issues were noted. Community Podoconiosis Agents (CPAs), who were all women, were expected by the group to conduct the more difficult tasks, such as going to more remote sites.

“This is [a] kind of small problem I have noticed for myself in the study area in Gojjam with building a team. I saw that in society and culture, where there is a difference between men and women, and where men are more dominant during screening. …. I saw … data collectors dominating the CPAs, [and] … women doing the hard work. … The cultural environment where the study is being conducted, you should really consider these things, because … in the end it will affect how people work, … it will affect how things are done.”–GoLBeT team member 2

The unbalanced workload in this case is seen as linked to the cultural environment.

The researchers recommended that, as part of trial leadership, these issues should be explicitly addressed and workloads should be balanced. The wider recommendation, based on this observation, is to consider the cultural environment in relation to how work is being conducted.

Further to experiences relating to the ethical approval process (discussed previously), proactive contact with the ethics committees was seen as useful, and generated good feedback. An example of such contact is actively inviting (and supporting) ethics committee members to attend a site visit; this is expected, but rarely realised due to time constraints.

For data management, the team recommended the use of electronic data collection devices, such as smartphones or tablets, in combination with OpenClinica. This included the importance of training, which was identified as essential at an early stage of trial planning.

## Discussion

### Recommendations

Reflecting back on the issues encountered during the trial set up, as reported in *Substantive issues*, and the *Reflections from the trial team* (*Results* section), five areas could be identified as crucial to its success: Collaborations, a team and process perspective, staff competency and training, community engagement, and data quality. They are summarized in Table 1, and will be discussed in more detail in the following.

**Table 1:** Summary of recommendations.

Area	Details	Tools
Initiation and maintenance of collaborations	National, e.g., NGOs, local health workers International, e.g., Kenya Medical Research Institute (KEMRI), Kenya Proactive contact with Ethics Committees	Support with project management, local knowledge and language, development of study design and statistics, SOPs, staff training, data management, backup server
Team and process perspective		Engagement of entire team early on Study walk-through Process evaluation using qualitative methods
Staff competency and training	Identify training needs early Provide training and support professional development	Global Health Training Centre of The Global Health Network Competency framework for capacity development (REF)
Community engagement	Cultural awareness Patient recruitment and retention Local language and dialects	Rapid Ethical Assessment (REA) Including local team members
Data quality	Data collection and data management documents/SOPs and systems	Electronic collection devices (smartphones or tablets) and appropriate software (OpenClinica) Patient diary design and testing Collaborations for advice and training

### Initiation and maintenance of collaborations

A clear recommendation that emerged from this methodology study was that collaboration with a variety of individuals and teams is important for the success of the trial. For example, the experience of the Principal Investigator, Co-Investigator and TSC helped to negotiate the approvals process, reducing delays. Their experience also helped the trial team to work on several set-up steps at once, which kept trial preparations moving. As discussed in *Study design* in the *Results* section, advice from the funders, groups at the KEMRI-Wellcome Trust programme in Kenya and the TSC was crucial for the study design. It led to a simpler design with one primary outcome, ensuring that the study was well powered to answer the research question. An initial lack of statistical help was a particular concern, and demonstrates to other trials the importance of seeking statistical expertise.

Local knowledge of health workers was also important in locating participants, helping to ensure that the required sample size was reached. The REA engaged members of the local community long before the start of the trial, ensuring that the trial was acceptable to the community. Piloting resulted in a well-designed, locally relevant patient diary that could accurately record acute podoconiosis episodes. Collaboration with other local organisations, such as NGOs treating podoconiosis, helped the trial team to standardise the definition, treatment and measurement of acute episodes. This may be important for other neglected diseases for which standardised definitions and procedures are not already in place. Proactive contact with ethics committees was found to be of advantage.

Finally, collaboration with the trial monitors in Kenya helped with training fieldworkers, developing SOPs, data management, and data quality. This partnership went beyond trial monitoring and contributed to capacity building for clinical research in Ethiopia.

“*In […] countries where there are few projects which are of the form of this randomised control trial*, *then the experience of Kilifi is very important*.*”–GoLBeT team member 3*

### Team and process perspective

The present study found that it is important to engage the whole team in the trial as early as possible, and moreover, that considering a process perspective is useful. In GoLBeT, the investigators worked with the operational team at an early meeting and used a ‘study walk-through’ process to discuss every element of the study with the whole team. This helped to make sure that everyone understood the research question, study design and protocol, and that all team members felt valued and knew their role was important. This walk-through process also allowed any potential areas of difficulty, such as operational and governance challenges to be pre-empted, and therefore mitigated through training and SOP development as needed.

### Staff competency and training

**A**mong the *Substantive Issues* in the *Results* section, staff recruitment was experienced as challenging due to a disconnect between qualifications/work experience and the required role in a clinical trial, according to defined role descriptions. This has been discussed before in the context of clinical trials run in low-resource settings [32]. Early identification of training needs was identified as crucial. Initiatives such as the competency framework for capacity development [33] and the Global Health Training Centre of the Global Health Network [34] are specifically aimed at providing support with recruitment and training of staff to groups undertaking health research, as well as their professional development.

### Community engagement

Interaction with the local culture, cultural awareness and local languages and dialects were seen as essential. REA was considered a very valuable tool for investigators, to facilitate collaboration with patients and their communities. This is of particular importance with regard to patient recruitment and retention, as discussed earlier in the *Results* section. The results of the REA have been published previously for GoLBeT [28]. For a different trial, REA was found to have a beneficial effect on informed consent comprehension in a low-resource setting [26]. Moreover, adapted to the context and seen as a form of stakeholder engagement, REA could serve as a useful procedure for making future pragmatic trials and, importantly, their results, more relevant for the target population. In a wider sense, REA should also be seen as a tool to understand the cultural context, and to develop appropriate strategies [28].

### Data management and data quality

As reported in *Data quality* in the *Results* section, ensuring data quality and setup of data management procedures were seen as crucial. Collaborations helped to ensure this goal in GoLBeT, for example through training, and help with design and set up of documents, for example SOPs, and systems, like the use of electronic data collection devices and appropriate software, such as OpenClinica. Local podoconiosis treatment groups advised. For recording of the primary outcome, a patient diary was designed including a local artist’s images of podoconiosis, and piloted and tested during a feasibility study. A clear motivation to collect high quality data was emphasized:

“At the end of the day, after the data [are] collected and analysed, we give, we inform policies, then we adapt. We exercise these policies for your brothers and sisters, right, and therefore people are inclined to collect [high] quality data.’–GoLBeT team member 3

### Regulatory procedures in pragmatic trials

Our research showed a lack of appropriate regulatory flexibility, as detailed in *Approvals and delays* in the *Results* section. This has been reported before in resource-poor settings [9], within Western sponsoring institutions, and within the clinical research community generally. Uncertainties regarding safety reporting procedures arose from an inability to adjust practice relative to the risk and complexity of the trial. This is likely to be because those involved had previously only experienced drug and vaccine trials with very different risks compared with a pragmatic trial, such as GoLBeT. More experienced research staff, and indeed reviewers on ethics committees, can adjust their operational and reporting plans to ensure that they are proportionate to the risks involved.

This study also suggests that official institutions in resource-poor settings may lack experience in working with clinical researchers, especially on randomised controlled trials. This may result in overinterpretation of the principles of GCP, delaying the approval of treatment trials that plan to use a safe intervention product. This has important consequences for pragmatic trials, for which the regulatory and reporting burdens are not the same as for a drug registration trial. If pragmatic trials are to be encouraged and supported, these burdens should be adapted relative to the risk of the intervention, and relative to the likelihood of consequential errors that would affect the safety and rights of the participants, or the integrity of the data [35,36].

### Conclusions

The original Global Health Research Process Map was built as a tool to guide the set-up of any clinical research study, as no similar tool existed. The aim was to help researchers to run studies of diseases and in regions for which evidence was lacking. It is an idealised map of the necessary steps and processes, designed to be as useful and applicable as possible for all settings. As was found for GoLBeT, the reality for any specific study will be more complex, and unlikely to follow a linear path. Therefore, operational methodology studies, such as the current analysis, are important to help us understand what happens in practice. Describing the realities of the set-up process for GoLBeT showed that steps were often interdependent, and that various steps needed to be thought about together in an ongoing process, rather than quickly accomplished in sequence. Many steps required a lot of ongoing thought, debate and decision-making, over an extended period. Steps sometimes needed to be revisited as more was learnt about the specific context of the planned study.

Overall, we hope that this report might guide others planning pragmatic trials in settings where research is not common, allowing them to anticipate possible challenges and address them through trial design, planning and operational delivery, and we hope that we could encourage such efforts.

### Strengths and limitations of the study

The action research approach chosen for this study and its interdisciplinary team ensured an atmosphere of trust between the GoLBeT trial team members and the social and clinical research scientists. This resulted in a collaborative atmosphere and quality control for analysis and interpretation, as well as the triangulation of findings.

At the same time, this resulted in a limited sample size for key informants who were consulted using the interviews or open-ended questionnaire, and the mini-group discussion. *Beyond sample size consideration*, *our study design and setup did not allow for an in-depth exploration of issues such as culture*, *ethnicity and gender in the project*, *in particular in the light of such a complex and multi-ethnic society like Ethiopia*. *Still*, *we believe these issues to be important here*, *and insufficiently addressed in the literature in general*. Potential limitations of the analysis specifically include usage of the Process Map as framework; however, we see the Process Map as a valid template as it was developed specifically for trials in LMICs. As a qualitative study, generalizability of results is not an aim. However, we consider transferability, that is, how well a study’s findings are able to inform health care contexts that differ from that in which the study was originally undertaken, and therefore applicability in different settings important [37–39].

## Supporting information

S1 Text GoLBeT initiation and set-up. Summaries of steps 1–41 of Fig 2.
(PDF)
