# Supplementary material for: Setting up a pragmatic clinical trial in a low-resource setting: A qualitative assessment of GoLBeT, a trial of podoconiosis management in Northern Ethiopia
Source: PLoS Negl Trop Dis. 2021 Jul 28;15(7):e0009582. doi: 10.1371/journal.pntd.0009582 (PMC8370641; doi:10.1371/journal.pntd.0009582)
Supplement: S1 Text — Summaries of steps 1–41 of Fig 2. (PDF) [file pntd.0009582.s001.pdf]

S1 Text: GoLBeT initiation and set-up - summaries of steps 1-41

1 – Develop Research Question
The Gojjam Lymphoedema Best Practice Trial (GoLBeT) for podoconiosis, in the highlands of Northern Ethiopia, was to be the first formal assessment of the impact of an intervention provided by local NGOs, before further expansion of this treatment program into neighbouring regions.

In September 2011, in the 'Case for Support' submitted to the funders (See 7 - 'Identify Source of Funding'), the principal research question was 'What is the impact of a community-based podoconiosis control program on clinical, social and economic outcomes in northern Ethiopia?'

Over time the objectives of the trial were developed from the initial research question. In the protocol, in December 2013, the primary objective was:

-To test the hypothesis that treatment decreases the frequency of acute attacks of podoconiosis

The secondary objective was:

-To test the hypothesis that treatment improves other clinical, social and economic outcomes

Hypothesised improvements in clinical outcomes included a reduction in the clinical stage of the disease, and a reduction in lower leg and foot circumferences. Hypothesised social outcomes were an increase in quality of life, measured with the 'Dermatology Life Quality Index', improvements measured with the 'WHO Disability Assessment Schedule', and a reduction in perceived stigma, measured using a scale developed for podoconiosis patients. Economic productivity was predicted to increase, measured by the number of days off work caused by podoconiosis.

2 – What is the intervention?
The intervention in the GoLBeT randomised controlled trial was a standardised version of a treatment for podoconiosis which was already being provided to patients by local NGOs. This treatment was pragmatic and involved the application of emollient, massage, bandaging and leg elevation to sufferers of this form of lymphedema.

Initially, in the February 2012 proposal to the funders (See 19 – 'Funding Proposal'), the study design was more complex: standard treatment was to be compared to a more intensive treatment. However, the intensive treatment was dropped, because it would have been difficult or impossible to roll out to a wider population after the trial, given the limited resources available (See 5 'Study Design').

The intervention needed to be standardised and this had challenges (See 8 – 'Intervention and Laboratory Organisation, Supply and Logistics' & also 15 – 'Develop Quality Assurance Plan'). There was a lack of formally standardised treatments in the literature for podoconiosis and also lymphatic filariasis – a disease with similar symptoms to podoconiosis. Different NGOs involved in treating podoconiosis were also using various intervention products, such

as different types of soap. However, due to the intervention being tested within a clinical trial, each stage of the intervention, such as the duration of leg elevation, had to be standardised. Discussions on standardisation of the intervention took place in November 2012, May 2013, and October 2013 when the Standard Operating Procedures (SOPs) were finalised.

3 – Characterise study and identify regulations

The GoLBeT randomised clinical trial was characterised as a research study because it was testing a hypothesis, had a clear research objective, and involved the collection of data that would not normally be collected as part of routine treatment for podoconiosis, as can be seen in the 'Case for Support' in September 2011 (See 7 - 'Identify Source of Funding'). Furthermore, GoLBeT allocated individuals to a treatment intervention, in order to evaluate the impact of treatment on health outcomes. Since GoLBeT was a clinical trial, the principles of ICH-GCP needed to be followed, after an analysis of the level of risk involved. It was necessary to register the trial to get an International Standard Randomised Controlled Trial Number (ISRCTN), and this was done online via the ISRCTN clinical trials website (See 25 – 'Register Clinical Trial'). Several levels of ethical approval were needed in Ethiopia (See 27/28 – 'Submit to Institutional Review Board(s)/Submit to Ethics Review'). Approval was obtained from the local School of Public Health, Addis Ababa University Institutional Review Board (AAU-IRB), and the national regulators: the Food Medicine and Health Care Administration and Control Authority (FMHACA), and the National Research Ethics Review Committee (NRERC) of the Ethiopian Science and Technology Commission (ESTC) (See 26 – 'Regulatory Approvals Submission'). Ethical approval was also needed from the Brighton and Sussex Medical School Research Governance and Ethics Committee, within the sponsoring institution in the UK.

4 – Develop study protocol

After the funding proposal was accepted for funding in September 2012 (See 19 – 'Funding Proposal'), this document was used at the basis for developing the protocol. Protocol templates were used during this process, and these were provided by a co-investigator at Oxford University, a collaborating institution with experience of running clinical trials. Researchers from organisations working on podoconiosis in the field were consulted on their practices so that the precise intervention to be used could be described in the protocol. An early version of the protocol was completed in November 2012, and used as a working draft. However, the protocol was developed further, after review by the Trial Steering Committee (See 33 – 'Steering Committee') and submission for ethics review, and amendments included changes to the trial objectives and inclusion and exclusion criteria, as seen in the version from December 2013 (see 11 - 'Finalise Protocol. Send to Sponsor' and also 23 - 'Sponsor Approval of Protocol').

5 – Study Design

The September 2011 'Case for Support' shows that GoLBeT was to be an intervention trial (See 7 – 'Identify Source of Funding'). Initially a 'stepped wedge' study design was considered. This is a type of Cluster Randomised Trial in which each cluster is randomly assigned to the intervention at a different time, and the effect of the intervention is measured each time, until all clusters eventually receive the intervention. This design means that only a relatively

small proportion of the participants are newly assigned to the intervention each time, and this resembled treatment practices for podoconiosis and was thought more ethical by the principal investigator.

'The stepped wedge in some ways gets round some of those ethical dilemmas and follows the natural rollout of a programme without say directly allocating individuals to immediate or delayed treatment. So it would've been slightly softer ethically, but much less robust statistically.' – GoLBeT Team member 1

Feedback from reviewing statisticians in December 2011 said that a conventional Randomised Controlled Trial would have more statistical power than the stepped-wedge and would be more straight-forward to analyse. The funders also requested reconsidering the stepped-wedge design in their feedback on the February 2012 funding proposal (See 19 Funding Proposal). Therefore, the stepped wedge design was dropped in February 2012 and an individual randomisation was chosen instead.

In February 2012 the funders also requested a clearer focus on one primary outcome. Initially there were several categories of primary outcomes – clinical, social and economic – and these were to be measured using a variety of tools. It was later decided it would not be possible to sufficiently power the study for each of these outcomes. Instead, after the funders' feedback, a single primary outcome was selected: the frequency of acute podoconiosis episodes (see 12 – 'Consider Study Population, Sample Size and Trial Statistics' and also 19 - 'Funding Proposal'). This was felt to be the most relevant outcome in terms of influencing clinical policy, because it could be accurately identified by patients and confirmed by fieldworkers

In July 2012 there was also a reduction from two to one treatment arm in the design, after further comments from the funders on the funding proposal. The initial design included an intensive treatment arm as well as the standard treatment, but the funders said this more complex design would not have had enough power to answer the basic research question. Furthermore, it was unlikely such intensive treatment would be provided to the population after the trial, given the resource-poor setting. The trial therefore compared standard treatment with delayed treatment as the control. The delayed treatment group received treatment a year later than the standard treatment group, after the effectiveness of the treatment could be shown (See 14 Risk Assessment). Further design issues in the GoLBeT trial involved blinding and randomisation. Blinding at the intervention site was not possible because it was obvious who was receiving treatment. However, blinding was possible and necessary for the purposes of randomisation, which was conducted after enrolment by statisticians at the clinical trials facility in Kilifi, Kenya, which was also monitoring the trial (see 20 'Peer Review'). Kilifi had no knowledge of who was being allocated to the treatment or the control and, since randomisation occurred after enrolment of subjects, the trial was unlikely to be influenced by selection bias.

6 - Identify Sponsor

Between February 2012 and October 2012 there was some debate over who would be the sponsor. The sponsor needed to be responsible for arrangements to initiate, manage, monitor

and finance the trial. The principal investigator who initiated the trial was based at Brighton & Sussex Medical School (BSMS) at the University of Sussex in the UK. It was established that the sponsor would be the University of Sussex. However, the principal investigator needed to apply to the Brighton & Sussex Medical School Research Governance and Ethics Committee (BSMS-RGEC) for sponsorship, at the same time as applying there for UK ethical approval. Sponsorship was then granted by BSMS-RGEC, allowing BSMS to confirm sponsorship on behalf of the University of Sussex.

7 – Identify Source of Funding

Between July 2011 and September 2011 the UK-based principal investigator discussed and planned for the joint MRC/DfID/Wellcome Trust call for Global Health Trials, with colleagues in the UK and in Ethiopia. The MRC is the UK's Medical Research Council, DfID is the UK government Department for International Development, and The Wellcome Trust is a global charitable foundation. This source of funding fitted the plan to evaluate the treatment program for the neglected tropical disease, podoconiosis. In September 2011 a preliminary proposal or 'Case for Support' was submitted to the funding bodies. In December 2011 this Case for Support was short-listed to go forward for a full application for funding (See 19 – 'Funding Proposal').

8 – Intervention and Laboratory Organisation, Supply and Logistics

GoLBeT was a pragmatic trial which needed the use of treatment centres. Only point-of-care diagnostics were needed, for screening for podoconiosis and ruling out a diagnosis of lymphatic filariasis. Options for intervention supplies were considered from September 2013 when the trial management team visited a different study in Mombasa and saw how intervention products were stored. On a visit to the Kilifi Clinical Trials Facility the trial coordinator and trial data manager were trained in the development of SOPs and logs (See 15 – 'Develop Quality Assurance Plan' and also 36 – 'Finalise SOPs'). SOPs were written and then translated for all aspects of treatment, including the exercise and leg elevation instructions which needed to be given to patients. However, standardising these was a challenge and required consultation of the literature and comparison of treatments given by different NGOs. The main intervention product was Whitfield's ointment and there were procurement discussions with the manufacturer in October 2013. GoLBeT also required shoes to be bought or made for patients and their sizes were to be measured during enrolment. Plans were also made for procuring other clinical supplies such as plasters and gloves. The local NGO was responsible for purchasing supplies, but the trial team facilitated purchasing, and this was very time-consuming.

'The one [organisation] who host it have their own administration but we help them to facilitate... to be fast, to be reached on the deadline, but that contribution was also very time-consuming, honestly.' – GoLBeT Team member 3

From November 2013 a substantial amount of time was also spent on deciding what information should appear on the labels for the intervention product: there were several drafts before the final decision in February 2014. This was then translated into the local language. A sample label was submitted to the national regulator, the Food Medicine and Health Care Administration and Control Authority (FMHACA).

9 – Develop Pharmacovigilance / Safety Reporting Plan

GoLBeT involved a publicly-available, non-experimental treatment, and between December 2012 and January 2013 safety procedures were developed and clarified in the protocol, whilst taking into account the low risks of the trial. An experienced co-investigator advised that directly using safety procedures from trials involving greater risk would not be helpful for GoLBeT because they would be unnecessary and cumbersome. Thus, monitoring and safety procedures were devised which were appropriate for the level of risk. For example, throughout proposal and protocol development, the co-investigator and principal investigator argued that a Data Safety Monitoring Board would not be needed (See 32 – 'DSMB'). The Trial Steering Committee (TSC) was initially unsure of safety monitoring and reporting for a pragmatic trial such as this, but in March 2013 it was concluded that expedited reporting would be required for Serious Adverse Events (SAEs) because the trial involved a clinical intervention. The GoLBeT protocol stated that health workers needed to report Serious Adverse Events (SAEs) within 24 hours to the Local Safety Monitor (LSM) and the investigators, and this arrangement eventually took the place of a DSMB, after formal agreement in the ethics review (See 27/28 – 'Submit to Institutional Review Board(s)/Submit to Ethics Review', and also 32 – 'DSMB'). If a SAE was further defined by the LSM and investigators as a Suspected and Unexpected Serious Adverse Reaction, this was reported within 24 hours to the Trial Steering Committee, Ethics Committees and Regulators. In September 2013, before the protocol had been approved in the ethics review, Standard Operating Procedures (SOPs) were developed for assessing and reporting protocol deviations and SAEs. The SOPs for safety reporting were used for Good Clinical Practice (GCP) training for data collectors and health workers (See 37 – 'Complete Training in SOPs and GCP'). In October 2014, due to the remoteness of the study, a full-time, on-site LSM was also recruited who could review SAEs quickly and report these to the Addis Ababa- based LSM.

10 – Finalise Intervention and Laboratory Requirements

Despite the GoLBeT trial using a publicly available intervention product, Whitfield's ointment, the Food Medicine and Health Care Administration and Control Authority (FMHACA) needed to approve the trial before supplying an approval letter to purchase the intervention product (See 26 – 'Regulatory Approvals Submission'). In October 2013, FMHACA had not yet approved the trial itself. However, it was still possible to discuss procurement options with suppliers whilst waiting for this approval. A local supplier was unable to produce a large enough quantity of Whitfield's in time for the trial and alternative pharmaceutical companies were approached. There were also attempts to obtain smaller and more affordable sizes of the intervention product, but this required the trial team to guarantee that they would continue to buy the product after the trial. In March 2014, after further discussion with FMHACA, it was decided that a one-off approval to purchase Whitfield's would be given at the same time that FMHACA approved the trial, and this approval was given in April 2014. A service agreement with the supplier was then signed in May 2014, with the shipment of the intervention product split into two batches. The first batch was due in June 2014, a few months before the expected start of the trial. The second batch was for delayed treatment and was to be delivered in October 2014, to allow for the relatively short shelf-life of the product.

11 – Finalise Protocol. Send to Sponsor

Finalising the GoLBeT protocol was a complex process involving review by various investigators and organisations. In January 2013 the draft protocol was submitted to the funding body. At the same time, this draft of the protocol was also sent to the sponsor, the University of Sussex, to keep them up-to-date on progress. After the formation of the Trial Steering Committee (TSC), the TSC reviewed and commented on the protocol between March 2013 and June 2013, helping to get it into shape before submission to the ethics committees (See 33 – 'Steering Committee'). The protocol was also amended on the basis of discussions after a trial team visit to the study site. Sample size was updated in the protocol and the main outcome measure was clarified (See 12 - 'Consider Study Population, Sample Size and Trial Statistics'). It was noted that the protocol had also appeared to suggest randomisation would occur before consent, which is contrary to Good Clinical Practice (GCP) and would have resulted in selection bias. The order of visits was therefore changed in the protocol, so that randomisation came after consent and enrolment. After this version of the protocol was approved by the TSC in June 2013, an ethics application was assembled and sent to Brighton & Sussex Medical School Research Governance and Ethics Committee (BSMS-RGEC), at the University of Sussex, and also to the ethics committees in Ethiopia (See 27/28 – 'Submit to Institutional Review Board(s)/Submit to Ethics Review''). Approval with recommendations was gained from the Institutional Review Board at Addis Ababa University (AAU-IRB) in December 2013. These recommendations required small changes to the protocol, such as the inclusion of withdrawal criteria. These criteria included when a patient wished to withdraw consent to participate, when a patient moved outside of the geographical area of the study, and when a patient experienced a Serious Adverse Event, compromising their ability to participate. These protocol changes were made in December 2013 and resulted in AAU-IRB approval at the end of January 2014. Further protocol amendments were also needed for regulatory approval, which was finally gained in July 2014 (see '23 Sponsor Approval of Protocol' and also '26 Regulatory Approvals Submission').

12 – Consider Study Population, Sample Size and Trial Statistics

The single study site of the trial was an area covered by an established podoconiosis treatment project set up by an NGO in 2010. In this highland area of Northern Ethiopia approximately 3% of adults are afflicted with podoconiosis. The study population for the trial consisted of adults of 18 years or older in the area, registered as having the disorder. Initially participants from the age of 16 were considered, but this was not permitted by the National Ethics Review Committee, who requested increasing the minimum age to 18 years. Health workers knew and were able to locate all adult patients in the area who needed treatment. In the mapping and screening process, data collectors visited these patients, gained consent to examine them at a later date, and mapped each patient using the GIS coordinates of their house (See 41 – 'Begin Recruitment').

February 2012 feedback on the funding proposal included a request to get statistical advice on the sample size, as well as advice on study design, randomisation and data management (See 19 – 'Funding Proposal'), including: selection of primary outcomes; estimation of effect size; number of intervention arms and number of confounders.

Primary outcomes

Advice from statisticians resulted in a change from several to just one primary outcome in order to ensure the study had sufficient statistical power. Previously several categories of primary outcomes were being considered: clinical, social and economic. It was decided to use incidence of acute podoconiosis episodes over 12 months as the single primary outcome, because this would be the most relevant outcome for clinical policy making. A quality of life index, which was previously an important measure for the social primary outcome was used as one of the secondary outcomes instead.

Effect size
Lymphatic filariasis is a disease with similar symptoms to podoconiosis. A lymphatic filariasis pilot study conducted by a different research group had suggested an effect size of 69%. The effect size of this pilot study was initially used to calculate sample size for the GoLBeT podoconiosis trial. However, in January 2012 statisticians thought that this effect size was likely to be smaller in the GoLBeT trial. In July 2012 the funders also requested a recalculation of sample size using a smaller effect size in funding proposal feedback (see 19 Funding Proposal). A reduction from 5.6 to 4 acute episodes of podoconiosis per year (effect size 28%) was eventually hypothesised and used to recalculate the sample size.

Number of intervention arms
The funding proposal of February 2012 stated that there would be a comparison of two active arms: standard and intensive treatment. However, in July 2012 the funders said that the trial would be unlikely to give a statistically significant difference between the two active arms. Thus intensive treatment was removed, leaving just one active arm, standard treatment, to be compared with delayed treatment.

Sample size calculation formula
In March 2013 the chair of the Trial Steering Committee noticed that the formula used to calculate sample size was for rare events only – one event a year or fewer. Acute episodes of podoconiosis typically number several per year and result in clustered data, due to within-patient correlations, which needed to be accounted for. However, it was found that the study still had enough power for the primary outcome without needing to increase the sample size. This was because the sample size had already been inflated to enable analysis for a range of potential confounders.

In April 2013, statisticians calculated the final sample size using a formula for the comparison of two means. For a standard deviation of 4.9, the sample size of 340 per arm gave 90% power to detect a hypothesised 28% reduction in acute episodes – from 5.6 to 4 episodes per year. This calculation included a 40% increase in sample size to take account of confounding variables, and also the 15% expected drop-out, given by the lymphatic filariasis pilot study. Due to a trade-off between the decrease in expected effect-size and simplification of the study design, these changes were not expected to result in any increase in costs.

A target of at approximately 2000 patients who were not yet receiving treatment were identified by health workers, located, and mapped. From these potential participants, it was planned that more than the 680 needed by the trial would be randomised, to allow for those who may not want to participate and those who were later found to not meet the inclusion criteria, such as a diagnosis of stage two or three podoconiosis. (See page 9, December 2013

protocol, for inclusion and exclusion criteria). It was not difficult to recruit, but the criteria meant that the number of neighbourhoods needed to be increased from five to nine in order to get a sufficient number of potential patients (See 41 – 'Begin Recruitment').

13 – Consider Data Management Requirements

The GoLBeT trial team started to think about data management requirements from January 2013, including the need to get the database and case report form (CRF) built. Technical equipment also needed to be considered. The Internet package was initially too slow and in March 2013 it was upgraded to a faster speed for the transfer of data. In May 2013 a high-specification laptop was procured for the data manager, including a 1TB external hard drive for backing up data. There was a close collaboration with the study monitors at the Kilifi Clinical Trials Facility in Kenya with regards to data management (See 15 – 'Develop Quality Assurance Plan' & also 20 – 'Peer Review'). OpenClinica was chosen as the software for data management (See 35 - Set Up Clinical Data Management System). In September 2013, Kilifi trained the Trial Coordinator and the Data Manager on using the software, as well as on the development of hard copy CRFs, Standard Operating Procedures (SOPs) and logs. Kilifi also provided training on GPS, which was necessary for mapping patients' houses, and in Mombasa the trial team observed the use of GPS and the completion of CRFs. In October 2013 data supervisors and data entry clerks were recruited and their technical needs considered. Because of the frequent power cuts in Northern Ethiopia, laptops were used on-site because they have a backup battery, and there was also a generator for backup power at the study site office. However, frequent loss of power at the study site required the data entry clerks to be located off-site in Addis Ababa, so that they could transcribe data onto OpenClinica templates without interruption.

'The main reason that we are making the data entry activities in Addis is due to the interruptions of this power, internet connections and other stuff like that. It is very, very, very difficult, about these power things and connections, in the study sites particularly.' – GoLBeT team member 3

For the same reason, the server was also set up in Addis Ababa, with the help of an IT specialist, together with a backup server at the Clinical Trials Facility at Kilifi in Kenya, which monitored the trial. An IP address was set up so that the Kilifi data manager and monitors were given the privilege to access trial data while still in Kenya.

14 – Risk Assessment

Discussions on risk assessment for the GoLBeT trial began as early as February 2012 while developing the funding proposal (See 19 Funding Proposal). The principal investigator argued in the proposal that a Data Safety Monitoring Board (DSMB) would not be necessary due to the low risks involved in the trial. The newly appointed Kenyan trial monitors (See 20 - 'Peer Review') also thought a DSMB would not be needed. These discussions continued throughout protocol development. The protocol itself was developed so that Good Clinical Practice (GCP) guidelines were adhered to without being over-interpreted, given the low risks. For example, it was ultimately agreed that a Data Safety Monitoring Board (DSMB) would not be required because interim data analyses would not be conducted and because adverse events were unlikely, and this was explained in the protocol of December 2013. However, since the trial

did involve an intervention, in place of a DSMB ethical committees and regulators required adverse events to be reported to a Local Safety Monitor. (See 32 - DSMB?).

There were discussions on intervention safety whilst developing the protocol in January 2013. Risks included adverse events from inexperienced bandaging, but these were to be reduced with training of health workers. Long-term use of the main intervention product, Whitfield's ointment, did not pose safety concerns because it was already publicly available and commonly used. In September 2013 a doctor experienced in the use of Whitfield's noted that the product does not encourage bacterial growth and is not likely to be absorbed systemically if swallowed.

A different type of risk was important to consider when developing Standard Operating Procedures (SOPs) with the Kenyan monitors in Kilifi, from September 2013 (See 15 – 'Develop Quality Assurance Plan' and also 36 – 'Finalise SOPs'). SOPs needed to be as short and simple as possible to limit the risk of study procedures being carried out incorrectly. It was also important for the trial team to know study procedures well to avoid discrepancies in documents. For example, the coordinator explained that a minor change to safety reporting, involving the recruitment of an on-site Local Safety Monitor in October 2014 (See 32 – 'DSMB'), affected the protocol, the SOP for the Local Safety Monitor, the SOP for SAE reporting, the Information Sheet, and the Consent Form. It was therefore necessary to know how such changes would impact on different documents so that they were consistent and did not result in protocol deviations.

'If one cannot identify what documents are affected, it will result in discrepancy. So, most important is to know study procedures very well; what happens when, where, by whom and what document is related to what.' – GoLBeT team member 2

A potential risk was that the trial would not recruit sufficient numbers of participants, due to patients mistrusting the trial and misunderstanding its aims. Early in the trial preparations, in January 2012, the principal investigator spoke about the need to spend time explaining the trial, the randomisation and delayed treatment process to the community. Therefore, after the recruitment of the trial coordinator and data manager in 2013, a Rapid Ethical Assessment (REA) and a sensitisation workshop were designed, to ensure the acceptability of the trial to the community. The REA took place in August 2013 and involved Focus Group Discussions (FGDs) with community members and patients, and In-Depth Interviews (IDIs) with experts and researchers. The REA showed that there was limited understanding of the difference between treatment and research.

'The REA indicated communities and patients have limited understanding on the differences between research and free treatment, they thought they're one and the same.' – GoLBeT team member 2

It also showed that randomisation and delayed treatment could be explained more clearly by using an agricultural analogy which was familiar to the community. In this analogy, a new fertiliser was used on selected fields, and yields were compared with those from fields with no fertiliser. After showing that the fertiliser was effective, it was supplied to all farmers. In GoLBeT, the control was the delayed treatment group, which received treatment a year after

the immediate treatment arm (See 5 Study Design). REA showed that patients did not mind being in the delayed treatment group because the effectiveness of the treatment would by that time be clearly shown, and they would be likely to have a much longer delay for treatment if they were not participants in the trial.

'This community had been waiting all their lives for treatment and if they go the business as usual and register with the NGO, then they're going to wait at least two years for treatment.' – GoLBeT team member 1

'Regarding delayed treatment, REA participants indicated people will not feel bad because it is like 'a God given chance and God doesn't take sides'' – GoLBeT team member 2

However, the method of randomisation was seen as important by the REA participants. Participants were concerned because they initially thought that randomisation would be done locally by trial staff who would know the participants, and that this would result in bias. The REA participants therefore demanded that randomisation be conducted in front of patients, so that they could judge whether it was a fair process. This is against Good Clinical Practice and was not allowed. However, when it was explained that the randomisation would be conducted in Kenya, by independent trial monitors with no knowledge of the participants, fears were alleviated to a large extent, even if they were not completely removed.

'They wanted it open, in front of them. But telling them that it was being done several hundred miles away in another country by people that weren't influenced by things going on in the community was a reassurance rather than the opposite. …it became less of an issue. I wouldn't want to say it's nothing at all, but it's less of an issue, yes.' – GoLBeT team member 1

The REA also showed how potential participants preferred to have information presented to them, the information needed, how communities felt about signing consent forms, and how patients would decide whether or not to participate. For example, participants wanted trial information to be presented to the community, and to be able to discuss the trial with their family and friends, before deciding whether or not to participate. It had been thought that potential participants might be fearful of signing informed consent forms because, elsewhere, property had been confiscated after people had signed documents they had not understood. However, GoLBeT participants were comfortable about signing the consent form provided that the trial was clearly explained. The REA findings were useful and in October 2013 they informed the design of the patient information sheet and consent form. These clearly explained the difference between treatment and research, and described the consent and randomisation process, alleviating barriers to consent to participate, and the forms were then sent for ethical approval (See 27/28 – 'Submit to Institutional Review Board(s)/Submit to Ethics Review'). A sensitisation workshop was also conducted with community leaders, including health workers, the local health office, local government leaders, educational leaders, and delegates from the agriculture office. This workshop involved presentations and question and answer sessions, and participants were briefed on the economic impact of podoconiosis, the significance of the trial, and the importance of collaboration for trial success. There was also a briefing on the differences between podoconiosis and lymphatic filariasis – a disease with similar symptoms – and the cause, prevention and treatment of podoconiosis, including the treatment given by the local NGO. The briefings also covered how

and why these villages and patients were chosen, the number of participants needed, what would be expected from participants, and the risks and benefits of taking part. The findings of the REA were also used to explain the randomisation process and why randomisation was necessary.

15 – Develop Quality Assurance Plan

Various measures were taken to ensure quality of data in the GoLBeT trial. In November 2012 a podoconiosis expert was recruited as a part-time Local Trial Monitor, to check quality. Team leaders and also data supervisors were to check that data collectors had correctly filled in questionnaires for the Case Report Forms (CRFs). The quality checking plan also included instructions for the trial data manager to check a random 10% sample of data transcribed onto OpenClinica templates, before it was uploaded onto the database. In June 2013 the approach for dealing with missing data and loss to follow up was discussed in relation to developing the analytical plan. Data collectors were trained in areas of Good Clinical Practice including data quality and also ethics. Central to quality assurance and training are Standard Operating Procedures (SOPs). SOPs were developed for data management, protocol deviations, Serious Adverse Events (SAEs), and for checking CRF entries and data queries. Most of the SOPs were based on templates or samples provided by Kilifi Clinical Trials Facility in Kenya from April 2013, a few months after it had been recruited to provide trial guidance (See 20 – 'Peer Review'). SOPs were developed in September and October 2013 with the help of Kilifi, when the trial coordinator and data manager visited the facility (See 36 – 'Finalise SOPs'). Kilifi also acted as the trial monitor and verified source data and its transfer to CRFs, thus ensuring data quality.

Other procedures also helped with quality assurance: for example, for consistency one person translated questionnaires in-house, whilst screening appointments were conducted in teams so that they could check clinical staging together. However, quality assurance became an issue with regards to the primary outcome for GoLBeT, which was to test whether there was a reduction in acute episodes – Acute Dermatolymphangioadenitis, or ADLA. There were difficulties in arriving at a standard definition for ADLA and its measurement (See 8 – 'Intervention and Laboratory Organisation, Supply and Logistics'). From March 2013 the literature for lymphatic filariasis was consulted because it is a disease with similar symptoms to podoconiosis, and there was very little literature on podoconiosis itself. However, various signs and symptoms were used in different studies and there were differences in how the number of episodes was measured. Furthermore, many studies used data from self-reported history, using local terms for episodes, and the accuracy of this was unknown. In May 2013 an experience sharing visit to an organisation with podoconiosis field experience helped to clarify how to define and measure acute episodes, but this still needed validation.

The ADLA diary was then developed for the patients to record acute episodes (See 35 – 'Set Up Clinical Data Management System'). In March 2014 a pilot study used focus group discussions to assess comprehensibility and acceptability of the diary. The diary contained pictures of podoconiosis drawn by a local artist, and it was confirmed that these were recognisable. This was followed by a feasibility study to verify that patients could understand and complete the diary over the period of a month, and to assess how long it would take to train patients in how to complete the diaries.

'…feasibility of using diaries in the GoLBeT trial among podoconiosis patients characterized by low-literacy to collect data on ADLA episodes was discussed and agreed it can be used successfully if clear instructions and images as well as information on when and how to complete diaries are provided to participants.' – GoLBeT team member 2

This feasibility study led to minor changes to the design of the diary. It also informed the instructions to be given to patients, which needed to emphasise that every day of the diary needed to be completed. Only a low proportion of the diaries were completed in the feasibility study because participants were not followed up. Therefore, in the trial, it was decided that all patients in the immediate treatment group would be prompted to bring their diary to be checked at monthly intervention appointments by health workers. Data collectors would also check a random 10% sample of control group diaries monthly, and they would check all diaries for both arms in the quarterly data collection visits (See p.12 in the December 2013 protocol). In May 2014, health workers were trained to identify acute attacks of podoconiosis, using a clinical assessment form, and they then conducted a diary validation study, verifying patient-identified acute episodes and noting discrepancies. Inaccuracies occurred because patients appeared to record *any* illness, but this could be rectified by emphasising symptoms specific to podoconiosis in later training. In the trial it was anticipated that patients would not always be able to complete diaries daily. In such cases the many religious days marked in the diaries were designed to help patients remember the beginning and end of acute episodes (See 35 Set Up Clinical Data Management System). This would allow data collectors to enquire about missing days and to complete any gaps in diaries retrospectively, during their visits. Diary completion was expected to require intensive supervision early in the trial, until participants were more familiar with procedures.

16 – Develop Trial Management System

GoLBeT did not have a Clinical Trial Management System (CTMS) as it was a relatively small, pragmatic treatment trial. However, the Trial Coordinator and also the Data Manager filed monthly reports which detailed key events, dates, recruitment, training of trial staff, and development of Standard Operating Procedures (SOPs), patient diaries and other documents. In addition the Data Management Plan described the process of day-to-day management of data, whilst logs recorded trial activities.

17 – Identify Insurance Required

In October 2012 there were queries within the GoLBeT sponsoring institution, the University of Sussex, about the process of getting insurance. This process was initially unclear for an overseas trial such as this. A co-investigator said that basic trial indemnity from the sponsor would be required, but wanted to steer the sponsor away from demanding a professional liability policy for staff. The Secretary of the Brighton & Sussex Medical School Research Governance and Ethics Committee (BSMS-RGEC) assumed that the University of Sussex would be insuring the trial and that an indemnity certificate would be issued by the university on approval of sponsorship. The issue of insurance was left until September 2013, when this certificate was written by an insurance officer at the University of Sussex (See 22 Organise Appropriate Legal Cover).

18 – Set Budget

A draft budget for GoLBeT was first produced in September 2011 with the Case for Support for the outline of the proposed research submitted to the funders (See 7 Identify Source of Funding). Changes to trial design in the February 2012 proposal revisions meant that the budget needed to be adjusted and queries from the sponsor regarding the budget table needed to be addressed, before resubmitting the proposal to the funders. One change was that the study duration was reduced, meaning there were sufficient funds within the original budget to recruit a data manager and trial monitor from the Clinical Trials Unit in Kenya to support the trial on a part-time basis (See 20 – 'Peer Review'). Changes to the study design and sample size that took place in July 2012 required the budget details to be revised again – these were attached as an appendix to the funding proposal, but the funders did not expect overall costs to increase.

A local NGO was to be responsible for carrying out the treatment and there were on-going discussions between the principal investigator and the NGO to ensure that the NGO's budget tied in with the grant administered by the sponsor, and the changes to the funding proposal. After the trial was approved for funding in September 2012, the details of the NGO's budget were revised in several rounds of amendments. In March 2013, the NGO was required to clarify how its costs were classified, to give more details to the sponsor on costs such as salaries, vehicles and travel, and explain discrepancies between their budget and the funding proposal. Internet costs were discussed with regards to the speed of service required. Costs were minimised where possible: for example, only basic GPS equipment was used for mapping, and data collectors had mobile phones and so didn't need landlines. Variable costs were discussed in October 2013, including recruitment of data collectors on fixed term contracts, who were paid by the day. The local safety monitor also needed extra compensation for adverse event checking. There were concerns around the NGO's lack of financial reserves, as is often the case for NGOs, and the potential for delays in expenditure payments to prevent research activities (See 38 – 'Are Study Supplies Ready?'). In GoLBeT there were delays in getting the NGO's initial budget paperwork in an agreed format, which resulted in delayed payments. Further delays in payments at the end of 2013 / start of 2014 were caused by the sponsor having a new finance system and because different levels of approval were needed before payments could be made. However, faster payment procedures were eventually set up.

19 – Funding Proposal

The funding proposal involved several submission stages. A preliminary proposal or 'Case for Support' was submitted in September 2011 and short-listed for a full application in December 2011 (See 7 - Identify Source of Funding). Submission of the full proposal in February 2012 was then followed by two rounds of comments from the funders, and responses to these comments, before the proposal was accepted for funding in September 2012.

In December 2011, the funders gave the go-ahead to submit a full proposal, but several queries needed to be addressed. More details were needed about the intervention package, the reliability of self-reporting and confirming acute episodes of podoconiosis, and generalisability of findings, and this information was added to the full proposal. The feedback on the preliminary proposal also recommended getting strong statistical advice on trial

design, sample size, data management, and randomisation. At the time, the medical school at the UK sponsoring institution was relatively new, and it had no Clinical Trials Unit and not many statisticians. The preliminary proposal suggested a stepped wedge study design but this was queried first by the funders and then by independent statisticians, known to the principal investigator, who were approached for advice.

A full proposal was submitted in February 2012, with attempts to reply to the funders' queries on the preliminary proposal. In response to the full proposal, the funding panel requested details of the sponsor, and so a letter showing approval of Sussex University sponsorship was written. The funders also requested a focus on only one primary outcome rather than several (See 5 - Study Design), and they asked for the stepped wedge to be reconsidered, because they were unconvinced by the arguments for using this design. Following further consultation with statisticians this initial design was rejected in favour of a conventional randomised controlled trial (See 5 Study Design). Work continued on the funding proposal budget table and a response to the funders' feedback on the proposal was submitted in June 2012. Further comments from the funders were then received in July 2012. The trial was said to be statistically underpowered and the funders requested a revised proposal and budget for a simpler design, a more modest effect size, and a recalculated sample size (See 5 - Study Design and 12 - Consider Study Population, Sample Size and Trial Statistics). This revised proposal was approved for funding in September 2012. The funders' comments on the proposal were helpful and resulted in a better-designed trial, more able to answer the research question.

20 – Peer Review

The preliminary proposal for the GoLBeT trial was short-listed in December 2011. However, the funding committee requested getting statistical advice before submitting a full proposal (See 19 – 'Funding Proposal'). The principal investigator sought advice on the trial design and statistics for the proposal, and this was an early form of peer review. In February 2012 the funders then requested stronger on-going statistical expertise and input, and collaboration with a clinical trials unit. A statistician at the clinical trial unit at one of the funding institutions was approached, but was unable to help because of institutional conflicts. However, a co-investigator suggested using the Kilifi Clinical Trials Facility in Kenya, which had an experienced data management group and statistician. Kilifi were interested in helping, and the use of this clinical trials facility provided a good example of capacity building which involved support from another African country. By this point, the GoLBeT design was simpler and shorter, because feedback from the funders had resulted in the stepped wedge design being dropped (See 19 - Funding Proposal). This simplification meant that in February 2012 a data manager and trial monitor from Kilifi could be recruited part-time within the original budget, providing on-going statistical help to the trial team in Ethiopia. Kilifi also acted as the trial monitor, helping to ensure data quality (See 15 – 'Develop Quality Assurance Plan'). Kilifi's involvement in monitoring and giving advice to the trial was invaluable since there was little experience of conducting randomised controlled trials in Ethiopia.

'…whenever there is a question, whenever there is a doubt, we ask the Kilifi staff by Skyping them.' – GoLBeT team member 3

Randomisation was also carried out in Kilifi (See 5 – 'Study Design'), and the GoLBeT data manager was able to learn from this experience.

'…the main thing is just to take the experience or to make capacity-building here. For instance, for the next randomised control trial, I am involved on the randomisation. …I want to learn more about the practical way of this randomisation.' – GoLBeT team member 3

The Trial Steering Committee was formed in March 2013 and this committee also played an important role in providing statistical input and peer reviewing the protocol (See 33 – 'Steering Committee').

21 – Secure Funding

The proposal was accepted for funding in September 2012 and an offer letter was received from the funders in November 2012. However, the trial team still needed to provide information in order to formally accept the offer. The funders needed to know which organisations would be approached for ethical and regulatory approval and when this would happen (See 27/28 – 'Submit to Institutional Review Board(s)/Submit to Ethics Review'). Details of trial registration were also requested. Details of the proposed Trial Steering Committee were provided to the funders. However, two of the proposed members were directly employed by collaborating institutions and were not considered sufficiently independent. All members of the final trial steering committee were approved in February 2013. The funders continued to request updates on ethical and regulatory approvals in Ethiopia and the UK (See 26 – 'Regulatory Approvals Submission' and also 27/28 – 'Submit to Institutional Review Board(s)/Submit to Ethics Review'). The funders also needed to be updated on further changes to the trial design, sample sizes, and the latest version of the protocol. Due to delays in gaining approvals (See 27/28 – 'Submit to Institutional Review Board(s)/Submit to Ethics Review') the trial started almost a year later than originally anticipated and screening did not commence until October 2014 (See – 41 'Begin Recruitment'). However, there was therefore little spending from the budget in 2014, and, in November 2014, the principal investigator was confident that the funders would agree to a no-cost extension to the trial, after explaining the reasons for the delay.

22 – Organise Appropriate Legal Cover

Enquiries about an insurance letter began in October 2012 (See 17 - Identify Insurance Required). This had not yet been provided for the trial in September 2013, when the trial team were preparing to apply for ethical approvals. The insurance letter was needed for the Study Master File, as requested by the study monitors, and for ethical approvals in Ethiopia. The University of Sussex had an insurance officer on the Brighton & Sussex Medical School Research Governance and Ethics Committee (BSMS-RGEC). The insurance officer advised that it was straightforward to issue an insurance letter, although this was not an automatic process. The officer drafted a simple insurance letter for the monitors and Study Master File at the end of September 2013.

23 – Sponsor Approval of Protocol

In July 2013, the GoLBeT trial was granted provisional approval by the Brighton & Sussex Medical School Research Governance and Ethics Committee (BSMS-RGEC). In February 2014, shortly after ethical approval from Addis Ababa Institutional Review Board (See 27/28 – 'Submit to Institutional Review Board(s)/Submit to Ethics Review'), BSMS-RGEC formally approved the trial and protocol on behalf of the sponsor, the University of Sussex. However, there were further protocol amendments during the submission process for regulatory approval, which was finally gained in July 2014. These were mostly minor amendments involving clarification of the treatment and use of the intervention product, but there was also a change in minimum patient age from 16 to 18 years old (see 26 - Regulatory Approvals Submission). A chronological list of protocol modifications was also needed for ethical and regulatory approval – this was sent to ethics committees with the updated protocol as required by Good Clinical Practice guidelines.

24 – Contracts Agreed

In late 2012 a contracts officer at the sponsoring institution requested details of collaborating institutions so that sub-contracts could be arranged. Details of key people at the Kilifi Clinical Trials Facility (KCTF) were needed in November 2012, although the sub-contract was between the sponsor and KCTF's partner, Oxford University. The legal status of KCTF's relationship with Oxford needed to be clarified for the sponsor in the financial agreement. In March 2013 the sponsor amended and finalised its contract with Oxford, and included the funders' guidelines for Good Clinical Practice, together with the protocol. The contract between the sponsor and the NGO carrying out treatment was discussed from December 2012. The NGO were keen to avoid cash-flow issues and initially wanted annual advances from the sponsor. A compromise was reached and a system of quarterly advances was formally agreed in April and May 2013, so that the NGO would still, in theory, always have funds for trial activities. The NGO needed to provide a study budget table showing a breakdown of estimated costs, at the start of each quarter, and this was to be approved and then paid by the sponsor. The NGO was then to send a financial report and invoice the sponsor for actual costs incurred at the end of each quarter, throughout the trial. If the advance for a quarter was greater than the expenditure, the surplus would credit the following quarter's invoice, and less money would be received for that quarter. If the costs were greater than the advance, the extra costs were paid by the sponsor. The contract between the sponsor and NGO was reviewed and agreed in June 2013, together with a grant letter from the funders. Whilst negotiations were ongoing for this contract, the principal investigator was able to fund the GoLBeT coordinator and data manager temporarily from another grant, allowing them to start work.

In January 2013 draft Terms of Reference (ToR) were drawn up for the trial coordinator and data manager, explaining their roles, and these were based on the trial management section of the Case for Support in the funding proposal. The ToR for the trial steering committee were drafted in February 2013, and these were based on templates provided by a co-investigator at Oxford University, who was experienced in managing trials (See 33 – 'Steering Committee'). In March 2013, in the first steering committee phone meeting the ToR were discussed and then approved. The ToR needed to be clear for less experienced members of the steering committee. It was clarified in the ToR that the role of the steering committee was advisory rather than trial management, and that the principal investigator had the final say on

decisions. ToR were also written for other investigators, such as the Ethiopian principal investigator in April 2013, and the local safety monitor in October 2013 (See 32 – 'DSMB'). Each ToR needed to be approved by the person carrying out the role.

25 – Register Clinical Trial
The funders required the GoLBeT trial to be registered, and for the registration details to be provided when the trial was accepted for funding in September 2012. The trial needed to be registered to get an International Standard Randomised Controlled Trial Number (ISRCTN), recognised by the WHO and the International Committee of Medical Journal Editors (ICMJE). This was done online via the ISRCTN clinical trials website. In January 2013 the names of ethics boards, approval dates, and reference numbers were requested as part of the registration process. These details were updated after gaining ethical approval and before the first participant was recruited. It was necessary to keep the funders updated on the ethical approval and registration process.

26 – Regulatory Approvals Submission
Ethiopian national regulatory approval for GoLBeT was needed from the Food Medicine and Health Care Administration and Control Authority (FMHACA) – now the Ethiopian Food and Drug Authority (E-FDA). In March 2013, there were discussions with FMHACA on gaining regulatory approval, which was needed despite the intervention product being publicly available. FMHACA also required approval from institutional review boards first (see 27/28 – 'Submit to Institutional Review Board(s)/Submit to Ethics Review'), and these were not received until January and February 2014. In February 2014 an application was made simultaneously to FMHACA and to the National Research Ethics Review Committee (NRERC) (See 28 – 'Submit to Ethics Review'). The applications required the trial protocol, as well as documents showing ethical approvals. Signed letters of collaboration with the School of Public Health at Addis Ababa University and with the NGO providing the treatment were also needed. FMHACA required minor clarifications in the protocol – for example, delayed treatment and the use of the intervention needed to be explained further. The minimum age was also changed to 18, despite having ethical approval from the Addis Ababa University Institutional Review Board for 16 and 17 year olds. FMHACA prohibits the use of children (people younger than 18) in randomised controlled trials in Ethiopia, and this can only be overturned by the FMHACA director in special cases. Permission to purchase the intervention product was also needed from FMHACA, assuring them that the product was only to be used for the trial patients. FMHACA approval was granted in April 2014, together with a one-off permission to purchase the intervention product.

27/28 –Submit to Institutional Review Board(s)/Submit to Ethics Review
Ethical approvals needed to be gained in a particular order for the GoLBeT trial – this section gives an overview of this process, incorporating ethics committees, institutional review boards, and the regulator.

GoLBeT needed ethical approval from three Institutional Review Boards (IRBs) and Ethics Committees (ECs): Addis Ababa University Institutional Review Board (AAU-IRB), Brighton and Sussex Medical School Research Governance and Ethics Committee (BSMS-RGEC), and the

National Research Ethics Review Committee (NRERC), and it was necessary to keep the funders up-to-date on progress. The trial team regularly followed up on the submissions.

Approval from the IRBs was needed before applying for regulatory approval (see 26 – 'Regulatory Approvals Submission'). Preparation for submission to the IRBs started with the development of the protocol (See 4 – 'Develop Study Protocol' and 11 – 'Finalise Protocol. Send to Sponsor'). The draft protocol from November 2012 (See 4 – 'Develop Study Protocol' and 11 – 'Finalise Protocol. Send to Sponsor') needed to be developed and then reviewed by the Trial Steering Committee (TSC) (See 33 – 'Steering Committee') to the point where it could be submitted to the IRBs. The TSC finally approved the protocol for this purpose in June 2013.

Ethics applications were made simultaneously to the department-level ethics committee within the School of Public Health (SPH), at Addis Ababa University, and also to BSMS-RGEC. The SPH quickly gave approval, also in June, whilst the BSMS-RGEC gave approval provisional on first getting approval from AAU-IRB. After gaining the SPH approval in June, an application could be made for faculty level approval from AAU-IRB. AAU-IRB gave full approval at the end of January 2014, resulting in the BSMS-RGEC confirming its full approval in February.
The application to BSMS RGEC also included questionnaires which were to be used at baseline, explaining that these were still in development and when they would be finalised. The patient information sheet and informed consent form were not yet drafted and it was explained to the BSMS-RGEC that these would be submitted for ethical approval after the Rapid Ethical Assessment (REA) (See 14 – 'Risk Assessment' and 41 – 'Begin Recruitment'). The BSMS-RGEC was prepared to wait for the REA to inform the writing of the information sheet and consent form, and it valued the REA process. At the end of July 2013 BSMS RGEC gave ethical approval provisional on also gaining approval from AAU-IRB. Unfortunately an internal miscommunication led to a delay before the application was sent to AAU-IRB, in October 2013. A list of protocol amendments and an updated protocol were sent to AAU-IRB. Drafts of the consent form and information sheet were also sent, with an explanation of delayed treatment, and the difference between research and treatment, which had been shown as important in the REA. There was some debate over whether to include participants younger than 18 in the trial. The AAU-IRB agreed to including 16 and 17 year-olds because the trial gave the prospect of benefits which would not otherwise be available to these children. However, the trial team later decided against this due to probable difficulties this would have caused gaining regulatory approval (see 26 – 'Regulatory Approvals Submission'). A further issue was that the AAU-IRB decided that they wanted a Data Safety Monitoring Board, something which the trial team had not thought necessary, although a compromise was later reached on this point (See 32 – 'DSMB?'). IRB approval was by now delaying trial preparations, and approval was still needed from the regulators. However, after some enquiries by the trial team, AAU-IRB gave approval with recommendations in December 2013. This required mostly minor changes, with some protocol amendments, such as the addition of withdrawal criteria. There were also revisions to the information sheet and consent form, with the addition of Rapid Ethical Assessment recommendations and international GCP requirements. The AAU-IRB also required English and translated copies of the information sheet, consent form, the Case Report Form, the Adverse Event reporting form, and screening and consent logs. Trial Steering Committee responses were also included in the resubmission to the AAU-IRB in late December 2013 (See 33 – 'Steering Committee'). The AAU-IRB then argued that the intervention is already in textbooks and needed to be convinced of the

importance of the trial. The principal investigator explained that the textbooks describe the intervention for lymphatic filariasis, and that there is no evidence other than a pilot study for the effectiveness of the treatment for podoconiosis. The AAU-IRB finally gave full approval at the end of January 2014, which resulted in full approval from the BSMS-RGEC in early February, and the start of the submission process to the regulators (see 26 – 'Regulatory Approvals Submission').

It was now possible to submit simultaneously to the regulator, the Food, Medicine and Health Care Administration and Control Authority (FMHACA), and also to the NRERC. The NRERC is an ethics committee within the Ministry of Science and Technology (MoST) and it helps MoST to oversee all science and technology research in Ethiopia. FMHACA gave approval in April 2014 (see 26 – 'Regulatory Approvals Submission'). However, problems arose with getting approval from the NRERC because their review committee and chair had been disbanded. In June 2014 the NRERC appointed a new chair. The regulator finally agreed to send the application to a single reviewer who was to be reappointed, followed by final approval by the new chair. Additional reviewers would not have been possible, because the rest of the committee had still not been reformed. In July 2014, the trial team responded quickly to NRERC comments and a translated version of the revised patient information sheet and consent form was also sent to the regulator. In the same month the NRERC gave their final approval, meaning that all approvals had now been obtained. Throughout the process the funders were kept up-to-date on the progress of the regulatory approvals.

The delay with gaining approvals had a knock-on effect, causing further delays due to the onset of the rainy season in Ethiopia. This meant that screening was not started until October 2014 (See 41 – 'Begin Recruitment'), and that there was an overall delay to the start of the trial of about one year, compared with the initial plan.

29 – Submit to Clinical Trial Authority Review

In Ethiopia the National Research Ethics Review Committee (NRERC) helps the Ministry of Science and Technology (MoST) to oversee all science and technology research in Ethiopia, including health research, and these are therefore similar to a Clinical Trial Authority (See 28 – Submit to Ethics Review). This can be contrasted with the Food, Medicine and Health Care Administration and Control Authority (FMHACA), which acts as a National Regulatory Authority (See 27/28 – 'Submit to Institutional Review Board(s)/Submit to Ethics Review', and also 26 – 'Regulatory Approvals Submission').

30 – Assemble Essential Documents in Study Master File

The trial team started to construct the Study (or Trial) Master File (SMF) in September 2013, two months after having gained provisional ethical approval for the trial from Brighton and Sussex Medical School Research Governance and Ethics Committee (BSMS-RGEC), and whilst developing Standard Operating Procedures (SOPs) with the help of the Kenyan trial monitors in Kilifi (See 15 – 'Develop Quality Assurance Plan'). It was not an easy process collecting together the many documents of the SMF, but the trial coordinator learnt a lot from the monitors, who also acted as mentors for the GoLBeT team.

'Again, it was not an easy process. I had to learn from trial monitors a lot. Now, I know what needs to be documented and where.' – GoLBeT team member 2

A copy of the SMF was needed at the office at the study site, while a second copy was needed by the sponsor at the time of the Site Initiation Visit (See 39 – 'Site Initiation Visit'). Various documents were needed in the SMF before the start of the trial:

- The latest version of the protocol, together with protocol amendments to date. The final protocol (See 23 – 'Sponsor Approval of Protocol') needed to document the agreement between the sponsor and the principal investigator
- Insurance letter from the sponsor – the University of Sussex – for basic trial indemnity
- Financial documents
- Agreements / contract with the NGO providing treatment
- Agreement / contract with Oxford University, on behalf of the study monitor at Kilifi in Kenya, including the Terms of Reference for the relationship between Kilifi and the trial
- Signed CVs and Good Clinical Practice (GCP) certificates for all investigators on the trial
- A copy of the Case Report Form (CRF)
- Informed Consent Form, Patient Information Sheet and translations
- Institutional Review Board and Ethics Committee members – names, qualifications and positions
- Dated ethics approvals, with all communications with Institutional Review Boards, and ethics committees, including applications & responses to comments
- Minutes of all trial-related meetings, including meetings with data collectors, and the agenda and minutes for Trial Steering Committee Meetings
- Investigator brochure for the intervention, with scientific information about the intervention product and the manufacturer's leaflet
- Communications / approvals from the regulatory authorities regarding the intervention product – the Food, Medicine and Health Care Administration and Control Authority (FMHACA)
- Translated sample labels for the intervention product
- Translated documents for patients and trial staff needed to be back-translated to check for accuracy, and signed translation certificates were needed for these documents

Building the SMF was an on-going process, which occurred at the same time the trial team was finalising the protocol, finalising and translating standard operating procedures (SOPs), and responding to Institutional Review Board and regulatory authority comments. The documents needed before the start of the trial were finalised around June and July 2014, after completing the Case Report Form, and after gaining the final approvals for the trial. Once the trial got underway in 2015 further documents were needed in the SMF:

- Signed informed consent forms
- Subject ID code list
- Subject enrolment log
- Intervention product accountability log

The intervention product accountability log needed to show that the intervention product was being used in accordance with the protocol. The log also needed to record intervention product purchasing, dispensing to treatment staff, and also quarterly reports with an inventory for the product.

31 – Setup Oversight Committees Stated in QA Plan

GoLBeT was a relatively simple trial with a publicly available and safe treatment. Because of this, full oversight committees were not always needed, or committees could play a multiple role, saving on resources. A Data Safety Monitoring Board was replaced by a Local Safety Monitor and a reporting procedure for Serious Adverse Events (See 32 – 'DSMB?'). The Steering Committee ensured that the trial could answer the research question, and it also served the function of the Protocol Review Committee (See 33 – 'Steering Committee'). The trial monitors helped to ensure data quality and also oversaw analysis and endpoint review (See 34 – 'Endpoint Review').

32 – Data Safety Monitoring Board (DSMB)?

Debate on whether a Data Safety Monitoring Board would be needed began in February 2012, when preparing the funding proposal. Trial team discussions with a co-investigator and with the Kenyan trial monitor questioned the role the DSMB would play. It was considered that a DSMB would not be needed, due to the low risk of the publicly available intervention (See 14 – 'Risk Assessment'), and an explanation of this was given in the proposal. However, after submitting to Addis Ababa University Institutional Review Board (AAU-IRB) for ethical review (See 27/28 – 'Submit to Institutional Review Board(s)/Submit to Ethics Review'), in October 2013, AAU-IRB decided that they wanted a DSMB. In response the trial team explained further why they thought a DSMB would not be necessary. Not only were safety events considered unlikely, but also GoLBeT was an open trial, meaning that the researchers and also the participants were aware of whether or not the treatment had been given. Furthermore, there was no interim data analysis to decide whether to end the trial early if the treatment was shown to be effective. Ending the trial early would have been inappropriate because there was not yet the capacity in Ethiopia to provide the intervention nationally. It was considered better practice to continue for the full 12-month treatment in order to get a complete set of data with good statistical power, which would make analysis clearer. Instead of a DSMB a Local Safety Monitor (LSM) was recruited to check adverse events, and this was a doctor with relevant clinical experience (See 9 – 'Develop Pharmacovigilance / Safety Reporting Plan', and also 14 – 'Risk Assessment'). This Addis Ababa based LSM was to visit the trial site four times a year and to immediately investigate any Serious Adverse Events (SAEs), reported within 24 hours by health workers to the LSM and to the trial investigators. This was explained in the December 2013 protocol, and AAU-IRB agreed to this. Much later, in October 2014 an on-site LSM was also appointed due to the remoteness of the trial site. This on-site LSM could review SAEs more quickly and report these to the LSM in Addis.

33 – Steering Committee

The Trial Steering Committee (TSC) played a central role, guiding the GoLBeT trial in reviewing and developing the protocol, improving the study design, giving advice on statistics and sample size, and gaining ethical approvals.

In November 2012, after the funding offer letter had been received (See 21 – 'Secure Funding) the funders requested a list of the proposed TSC members, together with TSC meeting dates, agendas, and minutes, as they became available. Unfortunately, two proposed members were not considered independent enough because of institutional conflicts: they were involved in the trial and employed by Oxford University and Addis Ababa University. Instead, a statistical epidemiologist from a different UK institution was recruited, and later an Ethiopian researcher with experience of podoconiosis. The epidemiologist had experience of clinical trials and became the chair of the TSC. The revised list of TSC members was sent to the sponsor as well as the funders, and the funders approved the TSC members in early February 2013. The TSC chair said it was standard practice to approve the Terms of Reference (ToR) for the TSC before going on to review the protocol. In March 2013, the ToR were emailed to the TSC in order to save time, since there were limited opportunities to meet. The TSC needed to be clear for members with less TSC experience. It was agreed that the TSC had an advisory role, with the principal investigator responsible for managing the trial, and this needed clarification in the ToR. The TSC was also to report to the funders and the sponsor on the progress of the trial.

The TSC meetings took place by Skype because the committee members were in different countries. In the first meeting, in March 2013, the TSC started to review the protocol, acting as the Protocol Review Committee, and this was important for protocol development and finalisation (See 11 – 'Finalise Protocol. Send to Sponsor'). The protocol needed clarification with respect to statistical issues, and these were addressed in this meeting. For example, the protocol needed to be clearer about the frequency of measurement in each trial arm, how missing data would be dealt with, the nature of the main outcome measure, and how contamination and bias would be avoided. The TSC chair also queried the sample size, commenting on the protocol after the meeting, and helped to deal with statistical issues (See 12 – 'Consider Study Population, Sample Size and Trial Statistics'), ensuring that the trial was well-powered. The revised protocol was sent back to the TSC in April 2013, including a new sample size and other amendments, and the TSC advised on how to report the sample size change to the funders. However, the TSC still wanted the protocol to be clearer on the primary outcome to be measured, and on the sequence of consent, enrolment and randomisation, before it approved the protocol in June 2013 for sending to the ethics committees. The TSC was updated on preparations for the trial in November 2013, in a second meeting/Skype call, and they were also sent the findings of the Rapid Ethical Assessment (See 14 – 'Risk Assessment' and also 41 – 'Begin Recruitment'), and a revised plan for the sequence of the enrolment process, so that they could comment on how to start the trial. The TSC was also involved in the ethical review process. In December 2013, Addis Ababa University Institutional Review Board (AAU-IRB) commented on the protocol and the trial team's responses required protocol amendments, such as the addition of withdrawal criteria (See 27/28 – 'Submit to Institutional Review Board(s)/Submit to Ethics Review'). The TSC asked to review these amendments and made further contributions to the protocol before it was resubmitted to AAU-IRB.

34 – Endpoint Review?

The primary endpoint in the GoLBeT trial was the cumulative incidence of acute podoconiosis episodes over the 12-month period following the start of treatment (See 12 – 'Consider Study Population, Sample Size and Trial Statistics', and also December 2013 protocol, p.7). The earlier work on standardising the measurement and recording of these episodes ensured that this endpoint was measured consistently (See 15 – 'Develop Quality Assurance Plan'). Consistency was made easier by the fact that there was just one study site. Furthermore, the study monitors in Kenya (See 20 – 'Peer Review') helped to ensure data quality and are to supervise data analysis when this is conducted in Ethiopia by trial statisticians (See December 2013 protocol, p.4). As of January 2015, the trial analytical plan had yet to be finalised. However, the statisticians will be blinded while they analyse the primary endpoint (See December 2013 protocol, p.9), and this will serve the purpose of an endpoint review committee. This blinding of the statisticians is particularly important in this trial because the GoLBeT intervention itself could not be blinded.

35 – Set up Clinical Data Management System

The Case Report Form (CRF) for each GoLBeT patient consisted of various source documents and questionnaires. The data was transcribed from these CRFs onto OpenClinica electronic templates, before being uploaded weekly to the OpenClinica database on the server in Addis Ababa, and the backup server in Kilifi, Kenya. Developing the source documents, CRF and database took time and a lot of thought to ensure that they were well-designed.

'…it takes to think a lot to read the different documents, to prepare the CRF and even the database.' – GoLBeT team member 3

The source document for the primary outcome was a diary kept by patients to record all acute episodes of podoconiosis (ADLA) (See 15 Develop Quality Assurance Plan). A first draft for this ADLA diary was created in June 2013 by the trial coordinator and developed through several drafts until January 2014, with the help of podoconiosis experts working locally. The aim was to keep the diary as easy to use as possible, with minimal text. Pictures of acute episodes were included, drawn by a local artist, and religious names and symbols were used as markers for days of the month, making the diary relevant to the local context. Each month of the Ethiopian calendar has different religious dates, meaning that the diaries for each month were different, and the logistics of providing these diaries needed to be considered.

In order to collect secondary outcome measures it was necessary to develop a clinical assessment form and socio-demographic and economic questionnaires, as well as validated translations of the Dermatology Life Quality Index (DLQI), the WHO-Disability Assessment Schedule II (WHO-DAS II), and a perceived stigma scale developed for podoconiosis. This was a team effort, involving the trial data manager, the trial coordinator and other investigators.

Work on the design of paper CRFs and the associated OpenClinica electronic templates began in September 2013, with the help of the Kilifi Clinical Trials Facility. A clear layout for the CRFs was important for data collectors and data entry clerks, and Kilifi advised simplifying the CRFs as much as possible so that they would be straightforward to complete. The design of the

CRFs was not finalised until June 2014, after work on the economic questionnaire and clinical assessment form had been completed, and permissions to use translated versions of tools such as the WHO-DAS II had been obtained. Since the design of the database was based on the paper CRFs the database design could then be updated. The need for data management Standard Operating Procedures (SOPs) and logs was also discussed with Kilifi. There were various SOPs, including for GPS, randomisation, data entry, and the data management plan (See 15 – 'Develop Quality Assurance Plan' and also 36 – 'Finalise SOPs'). Logs included CRF movement, data clarification, data query, recruitment, and Serious Adverse Events (SAEs).

36 – Finalise SOPs

In September 2013 the trial coordinator and data manager spent two weeks developing Standard Operating Procedures (SOPs) at the Clinical Trial Facility in Kilifi, Kenya, which was advising as well as monitoring the trial team (See 20 – 'Peer Review'). The team were able to develop most of the GoLBeT SOPs by adapting Kilifi's own SOPs, although it was not an easy process to finalise and translate them.

'Especially the translation of SOPs/ documents for use by fieldworkers in such a way it follows SOP models as well as to be easily understood by non-English speaking fieldworkers, was tough… …whoever plans to design study SOPs/logs has to clearly know the study procedures, consider who's to use them i.e. the end users and keep it short and simple understandable.' – GoLBeT team member 2

As for all documents, version control was important and a straight-forward system was needed to show changes to SOPs. A relatively minor change in one SOP could affect several other SOPs and documents, including the protocol, and the trial team needed to be very familiar with all study procedures so that documents remained consistent (See 14 – 'Risk Assessment').

The following types of SOPs were developed:

- Description of the Rapid Ethical Assessment (REA) process – including methodology & findings
- Community Engagement – from beginning of engagement to feedback of results
- Informed Consent process
- Mapping & GPS machine operation
- Screening & enrolment process
- Diagnostic test to rule out lymphatic filariasis – a disease with similar symptoms to podoconiosis
- Randomisation process
- Monthly Intervention Visits – including health facility & home visits
- Purchasing & transportation of materials by the NGO
- Intervention / treatment procedure
- Handling the Intervention Product
- Measurement & clinical staging
- Local Safety Monitors (adapted from another study)
- Reporting SAEs, protocol deviations, & hospitalisations

- Data Management Plan
- Data Collection
- Checking questionnaires, database entries, & CRF entries
- Query resolutions / checking data queries
- Endpoint measurement – Acute episode assessment; Patient diary completion; Diary verification; Questionnaire administration

The SOP for monthly intervention visits needed to be discussed so that these visits were well-structured and able to deal with non-compliance. Monthly intervention visits occurred at health facilities within two hours of patients' homes and allowed health workers to demonstrate treatment, ask about Adverse Events, record compliance, and issue treatment supplies. The SOPs for the intervention procedure and for handling the intervention product needed to have clear instructions: this was a challenge because of the lack of a standardised treatment for podoconiosis. The literature was consulted in order to standardise treatment and clarify these SOPs (See 8 – 'Intervention and Laboratory Organisation, Supply and Logistics'). All SOPs were sent to Kilifi for review and the experienced trial facility made important suggestions which greatly helped SOP development. However, this feedback needed to be assessed for its relevance to the GoLBeT trial, when translating SOPs for local trial staff, for example. In these translations, it was sometimes challenging to follow the Kilifi model whilst remaining easy to understand by fieldworkers. When translating the consent process SOP, the trial coordinator left out aspects which were not important for the local health workers – thus this SOP was simplified from the Kilifi model. By October SOPs for fieldworkers had been approved and translated, and were used for training purposes. Other SOPs took longer, but by January 2014 the remaining SOPs had been reviewed by Kilifi, finalised, approved by the principal investigator and then translated. However, there were further changes to SOPs when the minimum participant age was raised to 18 in March 2014, and when an on-site Local Safety Monitor was recruited in October 2014.

37 – Complete Training in SOPs & GCP

Discussion on the training of data collectors and health workers began in September 2013 and involved key GCP areas such as ethics, informed consent, confidentiality, data quality, and safety reporting. The training needed to emphasise the importance of showing respect for patients who were already stigmatised through having podoconiosis, and trial staff were to be encouraged to explore their own attitudes to the disease. Data collectors and health workers were also to be trained on the use of the protocol to guide all activities concerned with the trial, although it was decided that sponsor responsibilities and randomisation procedures did not need to be covered. GCP guidelines were consulted to check that other important areas would not be missed in the training.

In October 2013 training materials for GCP were prepared and translated, and the final SOPs for fieldworkers were also translated. These SOPs were as short and easy to follow as possible for the fieldworkers (See 36 – 'Finalise SOPs'). Also in October, the trial data manager recruited and trained health workers, data collectors, and data supervisors. Recruitment of suitable staff was often an issue because prospective fieldworkers often did not have the right experience, or those recruited were often overqualified for tasks such as data collection, which did not require a degree according to the data manager.

The training lasted for five days and was thorough and comprehensive, involving each SOP, data collection tool, questionnaire and trial log, as well as the principles of GCP. Trial staff received certificates in GCP after this training. In January 2014 GCP certificates and CVs were also received from all investigators on the trial.

The data manager described recruitment and training of fieldworkers as a challenge and this was not helped by the delays to the start of the trial (See 27/28 – 'Submit to Institutional Review Board(s)/Submit to Ethics Review'). In September 2014, data collectors and health workers needed to be retrained using the same five-day course, because of the year-long delay. Furthermore, some fieldworkers had been lost over the previous year and replacements needed to be found and trained. However, the data manager expected this type of additional work when working on a clinical trial.

38 – Are Study Supplies Ready?

Between July 2013 and January 2014 there were several potential delays in getting supplies ready. Delays in financial transfers from the sponsor to the local NGO conducting the treatment resulted in cash-flow issues and potential delays to procurement and field activities (See 18 – 'Set Budget'). In addition, intervention products such as soap and socks did not have records of shipment and importation, which the trial was required to document. There was also a concern about a shortage of Immunochromatographic Test (ICT) cards, which were needed in the screening to rule out a diagnosis of lymphatic filariasis, but this did not prove problematic. By March 2014 the start of the trial had been delayed instead by ethical and regulatory approvals (See 27/28 – 'Submit to Institutional Review Board(s)/Submit to Ethics Review'), but the office and general medical supplies were in place. It was decided that trial supplies should be partitioned off within the study site and it was stressed that the local NGO must not use trial supplies for general treatment purposes. In June 2014 the first batch of the intervention product was received and stored ready for the start of the trial, and the principal investigator visited and led a check on the facilities and supplies. Delays meant that screening did not start until October 2014, and the study supplies were checked again at the site initiation visit in December 2014 (See 39 – 'Site Initiation Visit').

39 – Study Initiation Visit

In November 2013, whilst waiting for ethical approvals (see 27/28 – 'Submit to Institutional Review Board(s)/Submit to Ethics Review'), the principal investigator (PI) and Kenyan trial monitor discussed the timing of the Study Initiation Visit (SIV). The PI thought that the SIV would take place after enrolment and randomisation, and at the time of patient initiation. However, the study monitor stated that the SIV should occur earlier, near the end of screening and just before enrolment began. The SIV would also spill over into the start of enrolment. This timing would allow the monitor to assess whether staff were prepared and familiar with trial procedures, and that the site was ready, before giving the approval for enrolment to begin. The monitor would also visit again later, to monitor and advise on the initiation of the treatment. The SIV was to be conducted by the trial coordinator, using the Standard Operations Procedures (SOPs), used previously for training, to 'walk through' the study with the trial staff (See 37 – 'Complete Training in SOPs and GCP').

In February 2014 the PI asked the trial coordinator when the SIV might occur, which would involve waiting for all approvals before starting the mapping and screening, as per GCP requirements, and then completing most of the screening before the visit. At the time it was estimated that all approvals would be gained at the end of April, that screening would be well underway in June and that the SIV could therefore occur in the second half of June. The PI visited the study site in June so that coordinator could show that the facilities were ready and that the trial could start quickly after regulatory approval, and after the SIV with the monitor. However, there were considerable delays and approvals were not gained until July 2014 (see 27/28 – 'Submit to Institutional Review Board(s)/Submit to Ethics Review'). The rainy season and the Ethiopian New Year meant that screening did not start until October and the SIV with the trial monitor did not occur until December (See 41 – 'Begin Recruitment'). The SIV took place over five days, from 8th to 12th December and the participating neighbourhoods and health clinics were assessed, together with the facilities, equipment and data management procedures of the coordinating site. During the SIV, the trial manager conducted the walk through of the study, supported by the Kenyan trial monitor, checking that all trial staff were ready to start the trial.

40 – Information Sessions for Study Staff

In GoLBeT the information sessions for the study staff occurred during the Site Initiation Visit (SIV) in December 2014, since the SIV took place immediately before enrolment (See 39 – 'Study Initiation Visit'). The SIV was conducted by the trial coordinator with support from the Kenyan monitor, and the operations manual, consisting of the Standard Operating Procedures (SOPs), was used to walk through the trial.

Earlier, in March 2014, the trial coordinator and data manager had explained to the health workers and data collectors that the delays to the start of the trial were due to the time taken to gain approvals. A more realistic timeframe for the start of the trial was discussed, and the trial staff were prepared to wait.

41 – Begin Recruitment

The sequence for the start of patient recruitment for GoLBeT was as follows:

- Screening / mapping – not to be started until all approvals obtained
- Site Initiation Visit (SIV) by the Kenyan trial monitor – near the end of screening
- Enrolment
- Randomisation
- Initiation of treatment

After gaining approvals in July 2014 the decision was made to postpone the start of screening and enrolment until near the end of the rainy season in mid-September and after the Ethiopian New Year. At this time the roads would be drier and transport would be easier. However, trial staff needed to be retrained (See 37 – 'Complete Training in SOPs & GCP') and screening slipped further, to early October 2014.

Screening carried on into November 2014 and this was a very busy time for the trial team, because they needed to visit study sites frequently. The trial team needed to increase the number of neighbourhoods from five to nine but the screening process succeeded in finding 850 patients who appeared to meet the inclusion criteria – although the screening test to rule out a diagnosis of lymphatic filariasis was not conducted until enrolment (See below). This number allowed for participants who may not want to participate or who may later need to withdraw, due to the inclusion / exclusion criteria, ensuring that after enrolment and randomisation there would be at least the 340 per arm required (See 12 – 'Consider Study Population, Sample Size and Trial Statistics'). The health workers involved in listing and mapping these participants knew all patients with podoconiosis in their neighbourhood because they had previously taken part in the governmental malaria and polio eradication programmes, and this local knowledge was very important to the success of screening.

'Therefore these health extension workers know each and every person around the kebele because they travel to the houses in the malaria eradication programme, in polio eradication programme by the government, then they know where the patients are.' – GoLBeT team member 3

The location of each patient's home was mapped by the trial team, whilst they accompanied the health workers during screening. Mapping involved recording GPS coordinates, and including landmarks such as churches, mosques and rivers, to help with patient location, although this mapping was less important than the local knowledge of the health workers.

The timing of the Site Initiation Visit by the Kenyan study monitors shifted to coincide with the end of screening (See 39 – 'Study Initiation Visit') in December. Enrolment then started immediately after the SIV, on December 13th, as it was important for the trial to get underway. The trial coordinator noted that not all patients were arriving on their enrolment date and said that this was due to the harvest season, with farmers busy in the fields. In addition, patients may have forgotten enrolment appointments. Early in the trial preparations, in February 2012, it was noted that the NGO providing treatment did not always report problems with non-attendance, which occurred during the harvest season and if a patient was far from the treatment site. To deal with this, in the December 2014 enrolment, the trial team sent data collectors and officials from the neighbourhood house-to-house to remind patients about their appointments. The enrolment visit was to go through the trial information a second time (after having first provided most of the information at screening), to gain full consent, and to collect baseline data. Enrolment also involved further screening to rule out a diagnosis of lymphatic filariasis, using an ICT test. The procedure for completing diaries was also explained at enrolment, and would be explained again during treatment visits (See 15 – 'Develop Quality Assurance Plan'). In December 2014, enrolment was expected to finish on around January 9th, 2015, and would be followed shortly after by randomisation by the Kenyan trial monitors in Kilifi (See 20 – 'Peer Review').

The earlier community engagement process, involving the Rapid ethical Assessment (REA) and the sensitisation workshop, was important for the success of recruiting participants and gaining a sufficiently large sample size (See 14 – 'Risk Assessment'). The trial coordinator noted that the REA greatly helped the design of the patient information sheet and consent form, and improved the trial team's understanding of the cultural norms of the community.
